# Functional insight into Maelstrom in the germline piRNA pathway: a unique domain homologous to the DnaQ-H 3'–5' exonuclease, its lineage-specific expansion/loss and evolutionarily active site switch

**DOI:** 10.1186/1745-6150-3-48

**Published:** 2008-11-25

**Authors:** Dapeng Zhang, Huiling Xiong, Jufang Shan, Xuhua Xia, Vance L Trudeau

**Affiliations:** 1 Centre for Advanced Research in Environmental Genomics (CAREG), Department of Biology, University of Ottawa, Ottawa, Ontario, K1N 6N5, Canada; 2 Department of Physiology and Biophysics, Weill Medical College of Cornell University, New York, NY 10021, USA

## Abstract

**Abstract:**

Maelstrom (MAEL) plays a crucial role in a recently-discovered piRNA pathway; however its specific function remains unknown. Here a novel MAEL-specific domain characterized by a set of conserved residues (Glu-His-His-Cys-His-Cys, EHHCHC) was identified in a broad range of species including vertebrates, sea squirts, insects, nematodes, and protists. It exhibits ancient lineage-specific expansions in several species, however, appears to be lost in all examined teleost fish species. Functional involvement of MAEL domains in DNA- and RNA-related processes was further revealed by its association with HMG, SR-25-like and HDAC_interact domains. A distant similarity to the DnaQ-H 3'–5' exonuclease family with the RNase H fold was discovered based on the evidence that all MAEL domains adopt the canonical RNase H fold; and several protist MAEL domains contain the conserved 3'–5' exonuclease active site residues (Asp-Glu-Asp-His-Asp, DEDHD). This evolutionary link together with structural examinations leads to a hypothesis that MAEL domains may have a potential nuclease activity or RNA-binding ability that may be implicated in piRNA biogenesis. The observed transition of two sets of characteristic residues between the ancestral DnaQ-H and the descendent MAEL domains may suggest a new mode for protein function evolution called "active site switch", in which the protist MAEL homologues are the likely evolutionary intermediates due to harboring the specific characteristics of both 3'–5' exonuclease and MAEL domains.

**Reviewers:**

This article was reviewed by L Aravind, Wing-Cheong Wong and Frank Eisenhaber. For the full reviews, please go to the Reviewers' Comments section.

## Background

Germline cells among different species are characterized by the presence of a morphologically unique organelle called the germ plasm (also referred to as nuage, polar granules or mitochondrial cloud) [1,2]. This organelle has been considered the determinant of germline development. Very recently a germ plasm-specific small RNA pathway has been identified, in which a new type of small RNAs called PIWI-interacting RNAs (piRNAs) or repeat-associated small interfering RNAs (rasiRNAs) play a role in ensuring the genomic stability of germline cells by silencing certain endogenous genetic elements such as retrotransposons and repetitive sequences [3-8]. Different from short interfering RNAs (siRNAs) and microRNAs which are usually 21–22 nt long, piRNAs or rasiRNAs have longer nucleotide composition (26–31 nt) and 2'O-methyl modification in 3' end. Many germ plasm proteins are functionally important in piRNAs synthesis and function, including PIWI proteins (PIWI, Aubergine and AGO3) [4,9,10], VASA [11], MAEL [11], SPN-E [12,13], Oskar [14], Tudor domain proteins [15], Armitage [13], Krimper [11], Cutoff [16], Dead end [17] and Zucchini and Squash [18]. Their loss-of-function mutations commonly cause a huge reduction in the amount of piRNAs or rasiRNAs and an increase in transcript level of transposable elements in the germline cells [11,19,20] as well as the spindle-class gene phenotypes: failure in establishing anterior/posterior polarity in early oocytes, disrupted asymmetric subcellular mRNA localization of Oskar, Gurken and Biocoid, ectopic expression of Oskar and Gurken, failure to proceed to the karyosome stage [8,11,13,21].

The molecular functions of most germ plasm proteins in the piRNA pathway have been assigned based on domain examination, biochemical and genetic characterizations. For instance, PIWI proteins contain the PAZ and PIWI domains, which contribute to recognition of single-stranded RNA [22] and sequence-specific endonucleolytic cleavage of target nucleotide [23,24], respectively. VASA, SPN-E and Armitage share DEAD RNA helicase domains, which provide helicase activities for piRNA production or retrotransposon silencing [13,25]. Zucchini and Squash are putative nucleases, which are believed to be involved in piRNA maturation [18]. Other Dead end, Krimper and Tudor proteins, contain RNA binding domains RRM [26] or Tudor [27] which may facilitate the assembly of multiprotein RNA-induced silencing complex (RISC) and targeting substrate RNA recognition during cleavage. In contrast, although many studies including specific knockouts, protein interaction and cellular distribution experiments have been conducted, the definitive function of MAEL in piRNA pathway remains unknown. MAEL was initially identified in a genetic loss-of-function *Drosophila *mutant, whose germline cells exhibit incorrect posterior localizations of several transcripts (*i.e*., Gurken, Oskar and Bicoid) [12]. It is a germ plasm-specific protein with all spindle-class gene phenotypes [12,13,21,28] and directly involved in the piRNA pathway [11,29]. The correct location of either SPN-E, VASA, Aubergine, Tudor or Krimper in germ plasm determines the location of MAEL [11], which in turn delineates the location of Dicer and Argonaute2 [21]. MAEL can shuttle between germ plasm and the nucleus [21]. Direct interaction between MAEL and chromatin remodeling proteins SNF5/INI1 and SIN3B during heterochromatin formation has also been demonstrated [30]. Therefore, MAEL is the only known protein connecting germ plasm and piRNA pathway to chromatin remodeling, a process required for piRNA-initiated genome transposon silencing [31]. In the present study, we were motivated to understand the putative function of MAEL using combined bioinformatic strategies including extensive homologous sequence mining, phylogenetic analysis, domain architecture, protein fold recognition, and structure modeling.

## Results

### A conserved MAEL-specific domain and its unique lineage-specific evolutionary expansion and loss

**Figure 1:**
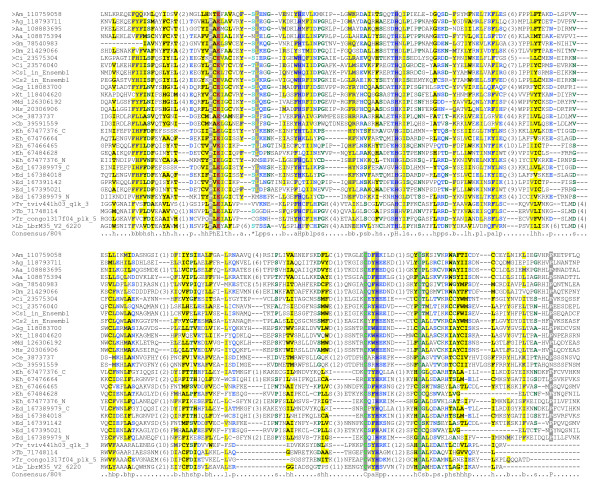
**Multiple sequence alignment of representatives of MAEL domain**. The sequences are represented by an abbreviation of species name followed by database entry ID. The homologues of *C. savignyi *identified in Ensembl database are indicated by Cs1 and Cs2. The consensus in 80% of the sequences is shown below the alignment based on default amino acid classes in Chroma. The numbers in bracket are indicative of the excluded residues from sequences. For a complete multiple sequence alignment refer to additional file 1. Species name abbreviations: Aa, *Aedes aegypti*; Ag, *Anopheles gambiae*; Am, *Apis mellifera*; Cb, *Caenorhabditis briggsae*; Ce, *Caenorhabditis elegans*; Ci, *Ciona intestinalis*; Cs,*Ciona savignyi*; Dm, *Drosophila melanogaster*; Ed, *Entamoeba dispar SAW760*; Eh, *Entamoeba histolytica*; Gg, *Gallus gallus*; Gm, *Glossina morsitans*; Hs, *Homo sapiens*; Md, *Monodelphis domestica*; Lb, *Leishmania braziliensis*; Tb, *Trypanosoma brucei TREU927*; Tr, *Trypanosoma congolense*; Tv, *Trypanosoma vivax*; Xt, *Xenopus tropicalis*.

Domain annotation showed that mouse MAEL protein contains a HMG domain in its N-terminal segment, which is a DNA-binding module in many non-histone components and transcriptional regulators [32]. However, no domain information could be assigned for the C-terminal segments of MAEL proteins (240 amino acids long). We conducted homologous sequence searching for this region using PSI-BLAST against the NCBI NR database. Many unique homologues were identified in a broad range of species from veterbrates, echinoderms, insects, nematodes, to the protists (*Entamoeba histolytica*, *Entamoeba dispar*, and *Trypanosoma brucei*). We also examined NCBI nucleotide and Ensembl genome databases and identified eight other homologues in insects and urochordates (*Ciona intestinalis *and *Ciona savignyi*). Three more protist homologues were obtained through searching GeneDB database. A multiple sequence alignment was built for all the retrieved sequences (additional file 1) and a condensed one is shown in Figure 1. Although the overall sequence identity is very low, the conservation is apparent across all these MAEL homologues. Six residues Glu-His-His-Cys-His-Cys (EHHCHC) are highly conserved, suggesting that they may contribute to MAEL-specific activity. Thus the C-terminal segment appears to define a novel MAEL-specific domain that we now refer to as the MAEL domain.

**Figure 2:**
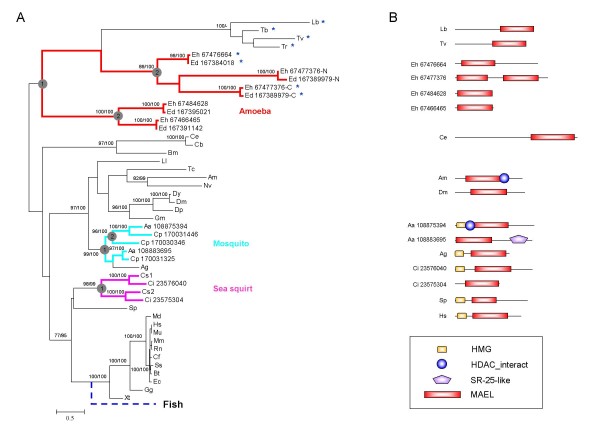
**Phylogenetic relationship and domain architectures of MAEL proteins**. (A) An unrooted phylogenetic tree was reconstructed using maximum likelihood (ML) analysis and Bayesian analysis. Single MAEL domains are represented by species names. The duplicated ones in the species of Aa, Ci, Cp, Ed, Eh are represented by species names following Genbank ID, whereas Cs domains are represented by Cs1 and Cs2. Branch length is proportional to estimated evolutionary change by PhyML program; the scale bar represents 0.5 substitution per site. Node supporting values greater than 75% from ML bootstrap analyses and Bayesian MCMCMC sampling are shown on the left and on the right of the slash, respectively. Lineage-specific expansions of MAEL domains in amoeba, mosquito and sea squirt are highlighted with different colors (red, turquoise blue and pink) and ancient duplication events were indicated by circled numbers. The loss of MAEL in teleost fish is indicated in blue dashed. Asterisk labeled MAEL domains are the ones containing both conserved EHHCHC and EDDHD residues (see following main text). (B) Domain architectures of representatives of the MAEL proteins were deduced through searching against Pfam and SMART domain databases and drawn approximately to scale. The domains shown are: HDAC_interact, named after Histone deacetylase (HDAC) interacting (SMART: SM00761); HMG, named after High Mobility Group (SMART: SM00398); SR-25-like (DUF1777, Pfam: PF08648). New species name abbreviations: Bm, *Brugia malayi*; Bt, *Bos taurus*; Cf, *Canis familiaris*; Cp, *Culex pipiens quinquefasciatus*; Dp, *Drosophila pseudoobscura*; Dy, *Drosophila yakuba*; Ec, *Equus caballus*; Lb, *Leishmania braziliensis*; Ll, *Lutzomyia longipalpis*; Mm, *Mus musculus*; Mu, *Macaca mulatta*; Nv, *Nasonia vitripennis*; Rn, *Rattus norvegicus*; Sp, *Strongylocentrotus purpuratus*; Ss, *Sus scrofa*; Tc, *Tribolium castaneum*. Other species name abbreviations refer to Figure 1 legend.

For the majority of species, only one copy of MAEL domain exists. However, there are multiple MAEL homologues in several other species; for instance, two copies are found in sea squirts (*C. intestinalis *and *C. savignyi*) and mosquito (*A. aegypti*), three copies in *Culex pipiens*, and five copies each in amoeba *E. dispar *and *E. histolytica*. Phylogenetic tree construction suggests that multiple MAEL copies are generated from a series of ancient lineage-specific duplication events (Figure 2A). Strikingly, no fish MAEL homologues could be identified. Its absence in teleost fish was confirmed by carefully examining the published whole genome databases in Ensembl for five different species (*Danio rerio, Gasterosteus aculeatus*, *Oryzias latipes*, *Takifugu rubripes *and *Tetraodon nigroviridis*). It can be inferred that it is the ancestor of the fish lineage after the divergence of teleost and tetrapod lineages that underwent the loss of MAEL domain. The timing of the loss is probably related to the ancient fish-specific genome duplication [33].

### Functional insight from domain architectures

Three other domains are associated with MAEL domains, including HMG (SMART: SM00398), HDAC_interact (SMART: SM00761), and SR-25-like domain (DUF1777, Pfam: PF08648) (Figure 2B). HMG is a common DNA-binding module in a variety of chromatin-associated proteins and functionally involved in the nucleoprotein complex assembly during genome recombination, initiation of transcription, and DNA repair [32]. The association between MAEL and HMG domains in most species suggests that the MAEL domain may somehow function in a DNA-related process. This functional assignment is also suggested by the association of MAEL domain with HDAC_interact domain in two homologues from mosquitoes (*A. aegypti *and *A. gambiae*). The HDAC_interact domain is known to bind to histone deacetylases (HDACs), core enzymes for removing acetyl group from lysine residue of histones during chromatin remodeling process [34]. It has been observed that pairs of interacting domains in one organism may have a fusion homologue composing of these two domains in another organism, known as the rosetta stone protein theory [35]. Mosquito MAELs may be rosetta stone proteins and it can be hypothesized that there are interactions between other MAEL and some HDAC_interact-containing proteins in other species. Indeed, it has been illustrated that mouse MAEL can interact with the SIN3B protein which contains an HDAC_interact domain [30]. The associated SR-25-like domain provides another link between the MAEL domain and RNA-related process. The SR-25-like domain is associated with RNA-binding modules, RNA recognition motif (RRM) [26] and PRP38 [36], It is also distantly related to SR-25 domain which may be involved in RNA splicing, as revealed by the SCOOP program [37]. Therefore, domain architecture suggests a potential involvement of MAEL domains in DNA binding, RNA binding and chromatin remodeling.

### A distant similarity between MAEL domains and the DnaQ-H 3'–5' exonuclease family with the RNase H fold

We applied a fold recognition strategy to identify remotely related homologues of MAEL domains. The rationale is that in the case of remote homology, conserved protein structural folds can be kept despite limited sequence identity [38]. A *meta *server was utilized, which assembles various state-of-the-art fold recognition methods and further evaluates modeled structures based on a consensus score computed by a 3D-JURY system [39]. MAEL domains from human, *X. tropicalis*, *Ciona *and *Drosophila *were first used as queries and several structural hits were identified by MetaBasic, ORFeus and BasicDist with consensus scores from 21 to 46. Although these 3D-Jury scores are below the cutoff 50, which corresponds to correct assignment with statistical significance [40], domain and fold examinations showed that all retrieved structures belong to the DnaQ-H 3'–5' exonuclease family with the RNase H fold [41,42]. We extended our search using an ancestral *E. histolytica *MAEL domain (GI: 67477376, residues 315–532) as a query. Eleven structural hits were identified with high scores around 58–69, and they all belong to the DnaQ-H 3'–5' exonuclease family. Structural fold similarities between DnaQ-H and MAEL domains encouraged us to re-examine this relationship using PSI-BLAST. We noticed that several DnaQ-H exonucleases can be retrieved as insignificant candidates in our initial PSI-BLAST searching with a profile inclusion expectation (*E*) value of 0.005. However, when we set inclusion *E *value at 0.05, significant similarity between the first 100aa segment of MAEL domains and several prokaryotic DnaQ-H exonucleases was achieved in the fourth iteration.

**Figure 3:**
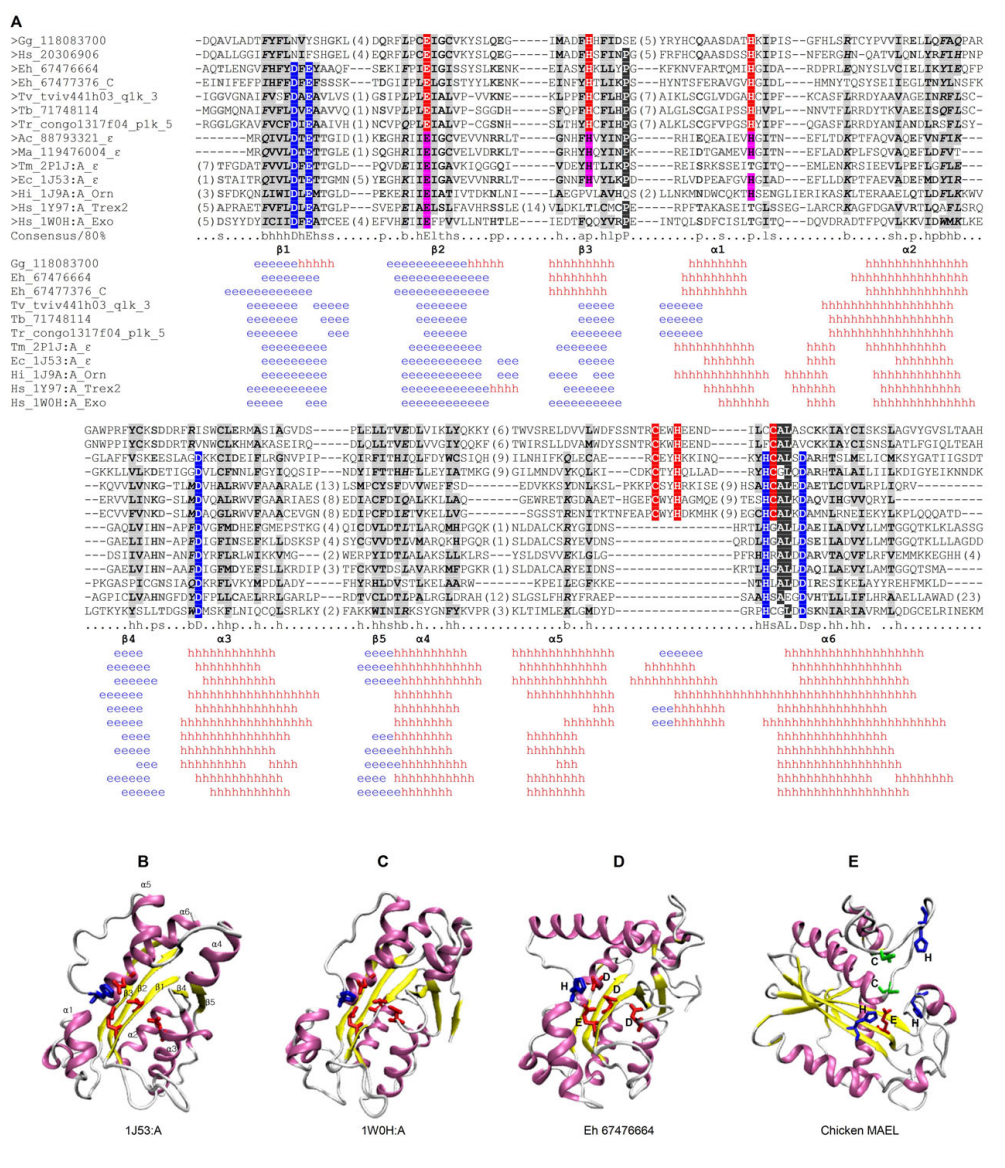
**Sequence and structure similarity between MAEL and DnaQ-H domains**. (A) Sequence and the secondary structure alignment of MAEL and DnaQ-H domains. Seven DnaQ-H domains are included and five domains have 3-D structures: *Thermotaga maritime* ε exonuclease (Tm ε, 2P1J:A),  *Escherichia coli *ε exonuclease (Ec_ε, 1J53:A), *Haemophilus influenzae* oligoribonuclease (Hi_Orn, 1J9A:A), human Trex2 exonuclease (Hs_Trex2, 1Y97:A) and human 3’-5’ exoribonuclease (Hs_Exo, 1W0H:A). The conserved DnaQ-H specific residues (DEDHD) are highlighted with the blue background; whereas the conserved MAEL-specific residues (EHHCHC) are highlighted with the red background. The MAEL-specific residues (EHH) also exist at counterpart positions in some DnaQ-H domains, and were highlighted with pink background. Detailed secondary structures for MAEL domains and DnaQ-H domains are obtained from secondary structure predictions and the 3-D structures, respectively; they are shown below the alignment (h in red, α helix; e in blue, β sheet). The structural sequence alignment was established carefully by hand on the basis of CE-MC results, alignment in fold recognition, literature information, and predicted secondary structures. The numbers in bracket are indicative of the excluded residues from sequences. New species name abbreviations: Tm, *Thermotaga maritime*; Ac，*Alteromonas macleodii*; Ma，*marine gamma proteobacterium*. (B-E) NewCartoon diagrams for DnaQ-H domains (1J53:A and 1W0H:A) and the homology model of two MAEL domains (Eh 67476664 and chicken MAEL). The α helices are shown in pink, β sheets in yellow, and loops in white; Their spatial locations are labeled in 1J53:A.  Strictly conserved DnaQ-H active site residues DEDHD of protist MAEL domain (Eh 67476664) are highlighted in licorice drawing with acidic residues (D and E) in red and basic His in blue. The MAEL-specific residues EHHCHC of chicken MAEL domain are highlighted with Glu in red, His in blue and Cys in orange.

We next examined this homologous relationship by building structure-based multiple sequence alignments for MAEL domains and DnaQ-H 3'–5' exonucleases. Since sequence identity among different DnaQ-H domains is very low, their alignment was first generated based on structural information as assessed by a CE-MC server [43] followed by manual adjustment based on published literature. Thereafter, we combined this alignment with the aligned MAEL domains based on fold recognition results and predicted secondary structures. The final alignment showed the conserved residues among/between two domains and compositions of secondary structures (Figure 3A). It is to be noted (Figure 3A) that equivalents of beta sheet (β) 3 of the RNase H fold in most MAEL domains are predicted to be alpha helix (α). We believe that this is a wrong prediction since in the canonical RNase H fold, β3 is an edge β strand, which can usually be misidentified as an α helix because of its solvent sequence property [44]. As shown in Figure 3A, the secondary structures of MAEL domains resemble those of DnaQ-H 3'–5' exonucleases; both have a β1- β2- β3- α1- α2- β4- α3- β5- α4- α5- α6 composition. More importantly, several ancestral protist MAEL domains also share all the critical DnaQ-H characteristic residues (Asp-Glu-Asp-His-Asp, DEDHD). These residues are commonly utilized by diverse DnaQ-H 3'–5' exonucleases and interact with two divalent metal ions to form an active site [45-47]. Thus, in contrast to a very low sequence identity (<15%) between MAEL domains and DnaQ-H 3'–5' exonucleases, the similar structural fold and the notable existence of DEDHD residues in protist MAEL domains strongly support a distant evolutionary relationship.

### Structural examinations on active sites by DEDHD and EHHCHC residues in MAEL domains

The tertiary structures of protist and chicken MAEL domains were further constructed by comparative modeling. Like DnaQ-H domains (Figure 3B, C), these MAEL domains adopt a similar RNase H structural fold which is characterized by a compact α/β fold with open anti-parallel β sheets in the middle and several α helices surrounded (Figure 3D, E). Moreover, the characteristic DEDHD residues in protist MAEL domains are clustered into a structural core, which resembles active sites of DnaQ-H domains (Figure 3A, B, C). In contrast, most other MAEL domains lack the DnaQ-H specific residues DEDHD. However, they are characterized by another conserved stretch of residues, EHHCHC. During evolution such conservation of MAEL-specific residues may reflect functional contributions most likely to a distinct active site. The spatial locations of EHHCHC residues were then examined in these modeled MAEL structures to check their possibility of forming an active site. Unexpectedly, we found that all MAEL-specific residues have very close spatial locations and they are clustered together at one side of the middle anti-parallel β sheets (Figure 3E). Four residues (EHHchC) can shape a structural core and other two residues may also potentially face down to it with slight structural rearrangements. Similar change of structural conformations of α5 and α6 comprising last CHC residues has been observed in crystal structures of DnaQ-H domains (additional file 2). There may exist another possibility that a disulfide bond (-S-S-) is formed between two Cys residues (C178 and C189 in the chicken MAEL domain) because of their close proximity. This is also supported by disulfide bond predictions [48]. Formation of a disulfide bond may facilitate the last His to approach other EHH residues, thus forming an active site with EHHH residues. Therefore, structural examinations suggest that protist MAEL domains with DEDHD residues may form a DnaQ-H active site whereas other MAEL domains with EHHCHC residues may potentially form a new active site based on the canonical RNase H scaffold.

## Discussions and conclusion

### Functional insight into MAEL in germline piRNA pathway

The proposed evolutionary link of MAEL domains to DnaQ-H 3'–5' exonuclease with RNase H fold may provide functional clues for MAEL domains. The DnaQ-H 3'–5' exonuclease family, also known as DEDDh exonuclease family or Exonuc_X-T domain (Pfam ID: PF00929), is one member of RNase H fold superfamily (SCOP: 53098) which also includes RNase H, mu transposase, crossover junction resolvase RuvC, and PIWI domain families [24,49-52]. They all share a canonical RNase H fold but contain different active site residues. The DnaQ-H family is characterized by five conserved residues, DEDHD, which form an active site in coordination with divalent metal ions (Figure 3A). Its members contribute to diverse nucleic acid metabolism processes such as replicative proofreading (1J53:A) [47], DNA repair or RNA degradation (exonuclease I and oligoribonuclease) [45,46], and RNA interference (ERI-1) [53]. Although different nucleotide targets (DNA or RNA) or diverse metal ions (Zn^2+^, Mg^2+^, or Mn^2+^) are involved [45-47], their active sites formed by the EDDHD residues delineates a common 3'–5' exonuclease activity. That is, the acidic DEDD together with two metal ions shape a negative pocket, which provides space for accommodating the 3' termini of oligonucleotide (DNA or RNA) chains. Thereafter, the coordinated metal ions and another conserved H are in direct contact with the bound chain, which induces a break of the phosphodiester bond of nucleotide in the 3'–5' direction [46]. Therefore, protist MAEL domains, harboring DnaQ-H specific DEDHD residues and active sites, may also employ a 3'–5' exonuclease activity, although their associated metal ions and nucleotide targets are still unknown.

In contrast to the protist MAEL domains, most recent MAEL domains do not contain the DnaQ-H specific residues but are characterized by the EHHCHC residues. What is the functional contribution of these residues to MAEL domains? Structural observations showed that a structural core can be potentially formed by the MAEL-specific residues EHHCHC or EHHH. This may provide a structural basis for an active site. On the one hand, this active site may confer RNA-binding ability for MAEL domains because of the lack of DnaQ-H specific residues. In this way, MAEL may contribute to stabilizing or positioning the RNA substrate in piRNA pathway. On the other hand, MAEL-specific residues and its potential active site may define another nuclease activity. We noticed that although all related families with the RNase H fold have low sequence identities and contain different active site residues, they all have DNA/RNA 3' or 5' end-directed nuclease activities with metal ion coordination in their own active sites [50,51]. For example, RNase H is a non-specific endonuclease whose catalytic activity requires divalent ions (Mg^2+ ^or Mn^2+^) and is responsible for the hydrolysis of the RNA in a DNA/RNA duplex [52,54]. In contrast, PIWI domains contribute to 5'-3' exonulcease catalytic activity for the Argonaute family proteins (Slicer) in all types of small RNA pathways (siRNA, miRNA, and piRNA). The activity is achieved by three PIWI active site residues, DDH, in coordination with one divalent ion and used to cleave single-stranded RNA substrate guided by complementary double-stranded small RNAs (piRNA or siRNA) [23,24,55-57]. It seems that the RNase H structural fold is an efficient scaffold from which diverse nuclease families have evolved distinct nuclease activities by developing their own active site residues with metal ion coordination. Therefore, being one member of RNase H superfamily, the MAEL domain may share this characteristic, thus the residues EHHCHC may form an active site with a new nuclease activity. It has been shown in diverse proteins that H, C and E residues often interact with Zn^2+ ^[58]. Moreover, the residue composition of EHHH is commonly utilized by several *Escherichia coli *proteins including ColE7 endonuclease [59], Zinc transport protein ZnuA [60], and Aldolase (1DOS) for their active sites, which also interact with metal ions, especially Zn^2+ ^[61],

Experimental evidence have suggested that MAEL may be involved in piRNA biogenesis since its loss-of-function mutant impairs the production of piRNAs or rasiRNAs and increases the transcript level of transposable elements [11]. Different from siRNA and miRNA pathways, piRNAs biogenesis employs a Dicer-independent mechanism [4,10]. A ping-pong model has been recently proposed for this process and it is hypothesized that AGO3 bound to the sense strand of piRNAs catalyzes cleavage of the antisense strand that generates 5' end of antisense piRNAs. The 3' end of the resulting antisense piRNAs is subjected to a 3' cleavage by an unknown endonuclease or exonuclease and a HEN1-processed 3' methylation. Thereafter, the produced antisense piRNAs associate with Aubergine or PIWI and direct cleavage of transposon sequences, which then generates the sense strand piRNAs after 5' cleavage, 3' cleavage and 3' methylation [5,7,8]. This cycling model is not complete since the exonuclease or endonuclease enzyme responsible for the 3' terminal maturation remains uncharacterized [5,7,8]. Thus, because of its evolutionary relationship to 3'–5' DnaQ-H exonuclease and the potential (3'–5' exo-) nuclease activities, MAEL may be the nuclease candidate implicated in the cleavage of the 3' termini. Recently, the nucleases Zucchini and Squash have been proposed as the 3' termini nuclease candidate based on the evidence that they are also located in germ plasm and have a similar mutation phenotype in a loss of transposon silencing [18]. However, MAEL is distinct from those above two nucleases due to its translocation between germ plasm and nucleus and the direct interaction with chromatin remodeling proteins [21,30]. We believe that multiple nucleases are involved in the diverse steps of piRNA pathway in a sequential manner, similar to PIWI family members targeting 5' cleavage of piRNAs [62]; and MAEL is involved in a genomic DNA-related piRNA step, which may include chromatin remodeling process and initial transcriptions of transposon. In this way, MAEL-associated HMG domain or other chromatin remodeling proteins facilitate the access of piRNA complex to the genomic regions where are enriched with transposon sequences. The transposon transcripts undergoing processing interact with the piRNA complex in which PIWI, one RNase H member, generates 5' end of transposon transcripts via a piRNA-directed homologous cleavage whereas MAEL, another RNase H member, contributes to a 3' terminal cleavage of transposon transcripts.

### Unique evolutionary characteristics for MAEL domains

Phylogenetic analysis has revealed several unique characteristics of MAEL domains including single-copy status in most species, ancient lineage-specific expansion and the loss in the teleost fish lineage. It has been long recognized that during evolution eukaryotic species have high duplication rates [63] and vertebrates have experienced two or three whole or regional genome duplications [33,64,65], which led to expansions of some domain families. It is of great interest that MAEL domain has escaped the usual duplication potential in most species, especially in vertebrates. It is also possible that the duplicated sequence was lost after duplication. However, it seems that this single-copy status is commonly inherited by several domains including SANTA domain [66]; an evolutionary selection against domain duplication together with the functional conservation, therefore, should account for the establishment of this status. We did observe MAEL domain expansion in several species. One or two duplication events occur at the ancestor of each lineage before its further divergence (Figure 2A). This ancient lineage-specific expansion may be caused by the release of evolutionary constraints in individual lineages. Thereafter, functional complexity may have arisen, as exemplified by diverse protist MAEL domains with either DEDHC+EHHCHC residues or EHHCHC residues (Figure 2A and legend).

We also observed the loss of MAEL domain in all examined teleost fish species. Gene loss in protein family evolution is well-recognized. The lost member may be functionally replaced by another member of the same family. However MAEL does not belong to this case because of its single-copy nature especially in the vertebrates. What happens in teleost fish germline cells without the MAEL protein? One possibility is that fish have a distinct but functionally similar counterpart, which remains to be characterized. Another possibility is that MAEL loss results in a unique piRNA pathway or a unique developmental morphology in fish germline cells compared to mammals and flies. Indeed, a distinct cellular distribution of Vasa protein, a marker for germline cells, has been observed in fish [10]. Moreover, it seems that although RNAi is evolutionarily conserved among species, individual lineage tends to develop some unique steps for the RNAi pathway, as shown in plant-specific XS domain in post-transcriptional gene silencing [67] and worm-specific Argonaute subfamily [62]. Furthermore, although the evolutionary and functional implications of MAEL loss in the teleost lineage are not yet understood, a practical implication can be hypothesized that fish may be amenable natural MAEL knockout-like models where transgenic insertion of MAEL proteins could be used to as a strategy for studying its function and the germline piRNA pathway.

### Active site switch, a novel path towards protein function change

**Figure 4:**
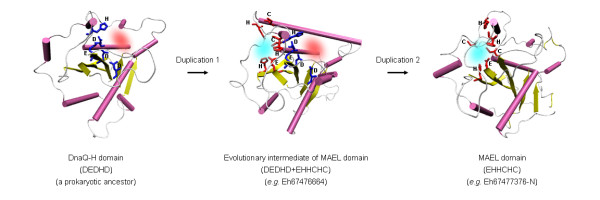
**A proposed "active site switch" mode for MAEL domain function evolution**. The cartoon drawing of protein structure is shown. The α helices are shown in pink, β sheets in yellow and loops in white. The DnaQ-H specific residues (DEDHD) are highlighted in blue, whereas MAEL specific residues (EHHCHC) are highlighted in red. Red cloud and green cloud indicate DnaQ-H active site and MAEL-specific active site, respectively.

How did MAEL domain evolve from the DnaQ-H domain? Considering the oldest identified MAEL domains are from *Protista *that represents the earliest eukaryotic branches [68], we believe that the first generation of MAEL domains should be traced back to an ancestral eukaryotic or a prokaryotic DnaQ-H domain, from which the MAEL-specific characteristics might have originated. Indeed, the first three MAEL-specific residues EHH are more ancient than others and commonly found in different prokaryotic ε exonucleases (Figure 3A). Their spatial locations are also close as shown in 1J53:A [47] (additional file 3), thus providing a substrate for evolving to a mature active site. It can be hypothesized that the DnaQ-H ancestor underwent a gene duplication event (additional file 4) in early *Eukaryota *or during the divergence of the prokaryotes and eukaryotes, corresponding to the time when small RNA pathways emerged. Thereafter, the duplicated one (MAEL ancestor) obtained a protein motif comprising CHC residues, forming an evolutionary intermediate which has both DEDHD and EHHCHC residues. The original DnaQ-H activity was attained by some ancestral protist MAEL domains. However, driven by relaxed evolutionary constraint associated with functional specification, other MAEL domains generated by further lineage-specific duplications or species speciation (duplication 2) may have lost the original active site with DEDHD residues, but at the same time developed a new active site with EHHCHC residues while keeping RNase H structural scaffold (Figure 4). The diversity of characteristic residues among three Eh MAEL domains (Eh67476664, Eh67477376-C, and Eh67477376-N) in an amoeba duplication branch (node value 89%/100%) supports this evolutionary path (Figure 2A). Compared to two other paralogs (Eh67476664, Eh67477376-C) which have both sets of DEDHD and EHHCHC residues, the Eh67477376-N has lost the DnaQ-H specific residues. Thus, MAEL domains have experienced a transition from DnaQ-H active site residues to MAEL active site residues which, we believe, may represent a novel mode for protein function evolution called the active site switch.

It has been long recognized that although protein superfamilies tend to preserve their structure during evolution, a divergent evolution with functional changes is permitted [38,69-71]. Protein function changes involve diversity or variability in active sites, properties of related residues or their spatial locations, as reviewed by Todd et al. [70]. Several possible mechanisms underlying protein function changes have been proposed including evolutionary optimization via functional residue hopping, independent recruitment of active sites in different lineages, circular permutation, and functional convergence after divergence [70]. Here, MAEL domains undergoing the active site switch provide another mode for protein function change; that is, during evolution new activities can be developed by introducing new active sites based on a preexisting protein scaffold. This evolutionary mode has long been hypothesized based on many *in vitro *directed evolution studies [72-74]. It has been shown that new activity can be introduced by simultaneous incorporation and adjustment of functional elements through insertion, deletion, and substitution of several active site loops, followed by point mutations to fine-tune the activity [73]. A similar process may have occurred in MAEL domain evolution. In addition, the ancestral protist MAEL domains which harbor the characteristics of both DnaQ-H and MAEL domains, for the first time, illustrate the existence of an evolutionary intermediate during protein function evolution. The identification of such an evolutionary intermediate may facilitate establishing real evolutionary links between protein superfamily members with different catalytic activities, or protein superfamilies which have overall similar structural folds but different functions.

## Materials and methods

See additional file 5.

## Competing interests

The authors declare that they have no competing interests.

## Authors' contributions

DZ initiated the idea, conducted data analysis and drafted the manuscript. HX and JS were involved in Bayesian phylogenetic tree construction and protein loop modeling, respectively. VT and XX contributed to discussion and revising manuscript. All authors have read and approved the final manuscript.

## Reviewers' comments

### Reviewer's report 1: L Aravind, National Center for Biotechnology Information, National Library of Medicine, National Institutes of Health, Bethesda, USA

Zhang et al show that the globular domain found C-terminal to the HMG domain in Maelstrom is a member of the 3'–>5' exonuclease superfamily of the RNase H fold. This finding leads to a key functional prediction that might help in understanding the role of this major regulator of gene expression which lies at the interface between the RNA-dependent process and chromatin dynamics. The basic relationship proposed here is sound; however, the authors note that the active site of this domain might have drastically been reconfigured in subset of the family with the utilization of an entirely new constellation of residues. This is a rather bold proposal based on homology modeling and the observed conservation. However, it is weakened by the fact that, as observed correctly by the authors, the canonical active site is preserved outside of the animal radiation along with the maelstrom family specific residues preserved in animals. This makes the claim suspect as it would imply that both active sites were simultaneously present in the ancestor. Hence, I strongly recommend that the authors completely rework this section and concede the strong possibility of the absence of nuclease activity in the forms lacking the canonical active site. It is quite possible that at least in animals it is an inactive RNA-binding protein.

### Authors' response

*Thank you for the invaluable comments. We have revised the whole paper to take into considerations these constructive criticisms. Towards the possible activity (either nuclease or RNA-binding) of MAEL with EHHCHC residues, we now only present structural evidence and give discussion based on other evolutionary and functional information. We agree with the reviewer on the question of whether both active sites (DnaQ-H and MAEL-specific activities) can be realized in the same protist MAEL domain. We believe they could since their conservation likely reflects functional contribution; otherwise the conservation of these residues should have been lost during evolution. It seems that because of the structural conformation constraints, the same protist MAEL domain cannot form two active sites at the same time; instead, it may adopt different conformations for each of the different activities*.

Further, there are several points in the paper that need to be addressed for it to be suitable for publication.

-Nomenclatural: Currently the authors use the term DNAQ-H for the superfamily. This is confusing as one could imagine that these indeed arose from DNAQ itself. Instead they should use the terminology "3'–>5' exonuclease superfamily in the RNAse H fold".

-Phylogenetic analysis: The resolution is probably insufficient and the topology of the tree appears suspect as a result. Further some proteins are evolving very rapidly and at different rates and distort topology (e.g. Entamoeba). The tree is not critical for the argument and it is best that it is presented in the additional file.

-Introduction is too long. The authors can very briefly state the importance of Mael and its biology rather than attempting the current detailed description.

-The key functional prediction and sequence/structure analysis can be also briefly presented.

-Rename DUF1777 to something more meaningful.

In conclusion a modified version of the paper, which is suitably condensed and presents the major findings succinctly would be suitable for publication as a discovery note in Biology Direct.

### Authors' response

*We thank the reviewer for these suggestions. We have revised the introduction, results and discussion sections and transferred the methods section to additional file *5. *We considered the proposal to put Fig *2A* in the supplement but feel strongly that it should be in the main paper. We discuss MAEL evolution extensively in the main text, so the tree will be important for readers to understand MAEL evolution, especially the transition between DnaQ-H and MAEL residues. We agree that some supporting values are weak, so we only present supporting value greater than 75% for the nodes. We renamed DUF1777 as SR-25-like domain because of its similarity to SR-25 domain family (pfam: PF10500) as revealed by the SCOOP program. For terminology, we used DnaQ-H 3'–5' exonuclease family with the RNase H fold. The reason why we emphasized DnaQ-H (also called DEDDh) is to differentiate another DEDDy family of 3'–5' exonuclease with the RNase H fold, which is characterized by conserved residues DEDYD*.

### Reviewer's report 2: Wing-Cheong Wong, Frank Eisenhaber, Bioinformatics Institute (BII), Agency for Science, Technology and Research (A*STAR)

In this paper, Dapeng Zhang et. al. attempt to decipher the molecular function of the germ plasm-specific protein, Maelstrom (MAEL) which has been implicated in the piRNA pathway and also in chromatin remodeling from previous experimental studies. The authors conjectured that Maelstrom has nuclease-like activity from three main findings: Firstly, the novel MAEL-specific domain is defined by a set similar sequence segments (related via a few PSI-BLAST searches) with a conserved motif involving residues (Glu-His-His-Cys-His-Cys) from mostly metazoans (except of fish species) and some protists. Some of these protist MAEL sequences also contain the DnaQ-H specific site (Asp-Glu-Asp-His-Asp) that exhibits a 3–5' exonuclease catalytic activity. Therefore, it seems likely that the metazoan MAEL proteins had inherited nuclease-like activity from their protist MAEL ancestors. Secondly, domain architecture analysis of MAEL-related proteins showed the association of the MAEL domain with the HMG (SMART: SM00398), DUF1777 (PFAM: PF08648) and HDAC_interact (SMART: SM00761) for DNA binding, RNA binding and chromatin remodeling respectively. Finally, structural modeling showed that the MAEL-specific domain (Glu-His-His-Cys-His-Cys) in metazoan is able to form a structural core despite the lack of the DnaQ-H active residues. The authors also argued that the residues His, Cys and Glu are the most frequently residues capable of interacting with Zn2+ and also utilized by ColE7 endonuclease, Zinc transport protein ZnuA and Aldolase; analogous to metal ion-binding DnaQ-H.

There are several critical points with this manuscript:

(1) The sequence segment family collection of homologous Maelstrom protein sequences is incomplete. Using the fan-like search methodology as described in Schneider et al. BMC Bioinformatics 2006 v.7, 164), more MAEL-like sequences including sequences from *Danio Rerio *(e.g. A2CF13_DANRE, EXOD1_DANRE,EXOD1_DANRE/Q502M8), oxidoreductases, DNA polymerases III and 3–5' exonucleases (e.g. Q503G0_DANRE, THEX1_HUMAN;1W0H:A) from numerous species can be found. Thus, there are homologs among fish species.

### Authors' response

*We thank you for your insights and suggestions. We also appreciate your attempts at retrieving additional sequences using your novel methodology. The sequences you identified all are DnaQ-H domains and some sequences you mentioned like 1W0H:A have been included in our study as representatives of the DnaQ-H domain. As we mentioned in the main text, PSI-BLAST searching with a profile inclusion E value of 0.05 can retrieve several DnaQ-H exonucleases as significant hits. However, they are not included in our initial sequence analysis for MAEL domains since they do not have MAEL specific residues (EHHCHC), and introducing these sequences may dilute conserved characteristics of MAEL domains. We used an E value of 0.005 for PSI-BLAST searches with different MAEL domains as queries. They all retrieved the same set of MAEL domain sequences. We also tried the HHsenser server, another sensitive sequence searching program, which retrieved similar results. We could not detect any fish MAEL domain from protein, nucleotide/EST or even Ensembl genome databases of five fish species. So, we are proposing that the MAEL domain is lost in fish species according to these observations. This discovery should be very interesting to experimental biologists who are working on the piRNA pathway. We agree that other distant homologs exist in fish, such as DnaQ-H members and other RNase H members (like PIWI)*.

#### Reviewers' response

The emphasis on a set of conserved positions (yet without a clear functional role) does not make the definition of a domain. Most importantly, the notion of globular domains unifies protein sequence segments having similarity of their fold (and, as a consequence, in their hydrophobic pattern). Besides understanding the types of protein families that are in the vicinity of the starting sequence, the purpose of performing fan-like search is also to determine if the search space of the starting sequence for its orthologous sequences is well sampled. When sequences are been collected, the relationship of orthology or paralogy is not obvious. But eventually, with sufficient sequence collection, sequences from different taxonomic groups will be able to form distinct group of protein families. Finally, with reference to these neighboring protein families, one can then use clustering or phylogenetic methods to determine the orthology coverage of the starting sequence. This has not been done in the work of the authors. We have carried out a full sequence family collection with a fan-like PSI-BLAST search (inclusion value for score matrix of ≤ 0.001; e-value for PSI-blast initialization < 0.06), aligned the family and created a phylogenetic tree from hits (with the group of exonucleases represented by the structure 1Y97 as outgroup, see attachment). It looks as if the so-called maelstrom group is surrounded by the bloom syndrome proteins (DNA helicases), DEAD-domain containing RNA helicases followed by bacterial nucleases as next hits. The fish sequences mentioned by us are in the neighboring helicase groups and, apparently, are not nucleases.

### Authors' response

*We have conducted profile-profile alignments between MAEL, DnaQ (Exonuc X-T, Pfam: PF00929), and DEAD helicase (including bloom syndrome proteins, Pfam: PF00270) domains using the logomat-p program (additional file *6). *In contrast to detectable similarity between MAEL and DnaQ domains, no global similarity between MAEL and DEAD helicase can be identified. The similarity between MAEL and DnaQ domains is shown for the first 100 amino acid segment, also seen in the PSI-BLAST results. The reason why the second half segment does not appear to be homologous is that conserved residues are different (CHC in MAEL and HD inDnaQ) and that no structural fold considerations were made. Therefore, the evolutionary tree inferred from unrelated sequences is not reliable. We do not agree with the assessment of the reviewers*.

(2) An exhaustive search for homologous sequences across all species is the foremost important task in function annotation transfer via homology. This exhaustive list of the homologous sequences enables one to construct clusters of orthologous and paralogous genes and to group them in a phylogenetic tree. Among orthologous sequences, function annotation transfer is able to hold well especially for one-to-one orthologs, with decreased confidence at greater evolutionary distances. On the opposite end, paralogous sequences are generalized to be functional diversified and specialized. This makes the task of function annotation transfer more complex (see Koonin, 2005, Annu. Rev. Genet., 39, 309–338). In this paper, the exact homology relationships among the collected sequences were not well established and, thus, function annotation transfer in this context is problematic. It appears to us that the exonucleases are in another branch of the tree compared with maelstrom sequences; thus, the predicted function might not be correct.

### Authors' response

*This paper presents an evolutionary relationship between MAEL domains and DnaQ-H domains with the RNase H fold based on structural fold similarity as well as the evidence that protist MAEL domains have DnaQ-H specific residues. We do agree that a direct function annotation transfer may not be guaranteed based on this evolutionary link because of functional divergences during protein evolution. But considering the general functions in nuclease activities of DnaQ-H family as well as its distantly related RNase-H superfamily members, we predicted that MAEL may have a similar function with either nuclease or RNA-binding activity. We provide a preliminary evolutionary tree between DnaQ-H and MAEL domains in the additional file *4* and combined this with Figure *2A* for extensive discussion in the last section. We hope this discovery will facilitate the further investigation on MAEL function*.

#### Reviewers' response

We think that the conclusion about the functional relationship to the DnaQ-H domain is premature in this form. A hit with 3D-jury is, at best indicative. Our family search and the resulting phylogenetic tree (see attachment) bring the maelstrom group equally close to various helicases and nucleases. This more stringent homology search results (inclusion value for score matrix of ≤ 0.001; e-value for PSI-blast initialization < 0.06) revealed that the Maelstrom sequences are in close vicinity to a group of Bloom syndrome proteins (belonging to the DNA helicase family), bacteria nucleases and helicases while the exonucleases were not significant enough to be found (consistent with authors' PSI-blast results of insignificant p-value for the exonucleases). A preliminary phylogenetic study (with exonuclease as the out-group) showed that the Maelstrom sequences are most homologous to the Bloom syndrome protein sequences in comparison to the other sequences. DnaQ-H is by far not the closest functionally characterized neighbors. In the absence of further structural and catalytic information of the MAEL motif (Glu-His-His-Cys-His-Cys), the functional evolution relationship between Maelstrom and exonuclease is still unclear except for a potential similarity of fold.

If you do not have an own resource for correct family collection, we strongly suggest the authors to use protein family searcher like HHsenser  to collect more homologous sequences to clarify the relationship of Maelstrom to its adjacent protein families.

### Authors' response

*As we indicated previously, no sequence similarity can be detected between MAEL and DEAD helicase domains. We tried HHsenser to retrieve MAEL homologues sequences, and it generated similar results as PSI-BLAST. We thank reviewers for this suggestion*.

(3) Furthermore, the suggestion of nuclease-like activity in metazoan MAEL proteins is weak given that the DnaQ-H active residues were not conserved even if the predicted tertiary structure is correct and, probably, conserved in the family. A structural is only a plausibility argument; it does not prove the conclusion. Doubts are the more appropriate since the homology model involves a translocation/shifting of the active site.

At the end, a set of sequentially similar sequence segments without any trustworthy molecular function prediction remains. This result is not necessarily demanding another publication.

### Authors' response

*We present general discoveries about MAEL, its evolutionary link to DnaQ-H domains and structural predictions on active sites. We agree that functional prediction is not the definitive conclusion. However, we believe that our rigorous analysis may give us a strong basis to hypothesize on function. Firstly, the evolutionary link and possible DnaQ-H active site in protist MAEL domains may suggest that protist MAEL domains have a 3'–5' exonuclease activity. Secondly, for the MAEL domains with EHHCHC residues, the high conservation of these residues likely reflects their functional contributions. Structural examinations direct our attention to an active site since these conserved EHHCHC residues are located closely together. We then found other evidence including the property of E, H and C residues to interact with metal ions and general functions of evolutionarily related RNase-H fold families. Although we do not have experimental support, these lines of evidence provide structural, chemical and evolutionary basis for an active site, and thus lead to our hypothesis that it may have nuclease activity or RNA-binding ability. Thirdly, translocation/shifting of the active site is common in evolution of protein families as reviewed by Todd et al. (2002) and Anantharaman et al. (2003). It is also true for the RNase H fold superfamily in which the DnaQ-H family and other families use their own specific residues to form different active sites. Therefore, we believe that the DnaQ-H active site is lost during MAEL evolution and the MAEL domain developed its own active residues. More importantly, we identified some protist domains which have both sets of active residues of DnaQ-H and MAEL domains. They can serve as an evolutionary intermediate during this translocation/shifting, thus suggesting a new mode for protein function evolution*.

#### Reviewers' response

Firstly, the authors utilized the 3D-jury results to indicate that the maelstrom protein segment might confer a similar fold to that of DnaQ-H domain exemplified by pdb 1W0H:A. It appears to us that the evolutionary distance to these exonucleases is considerable and that other groups are much more closely related. We found the Maelstrom sequences to be most homologous to the Bloom syndrome protein sequences. Therefore, the structural fold prediction might not be reliable and, at such evolutionary distances, it would be not surprising if the relative positions of important residues are scrambled. Furthermore, the metazoan Maelstrom proteins have lost these residues that appear indispensable for the nuclease activity. Unless it can proven experimentally, the suggestion that metazoan Maelstroms have nuclease activity seems less plausible, especially given the presence of a more closely related group of Bloom syndrome proteins.

Secondly, Anantharaman et al. state that the presence of a characteristic set of conserved active residues is important for the identification of enzymes in sequence analysis. The set of conserved active residues are typically derived from known set of sequences and structures of related enzymes. For those proteins with preserved structure but varying catalytic residues, the detection of evolutionary relationship is far more difficult. In the case of the Maelstrom, the structure is purely hypothetical and the MAEL motif (Glu-His-His-Cys-His-Cys) has yet to show nuclease-like activity. Therefore, to say that a translocation or shifting of the active nucleatic site has occurred in the Maelstrom in the course of its evolution simply cannot be proven at this point without further experimentation or other type of compelling information. Thus, the molecular function of the maelstrom domain remains unclear and the current stage of research does not justify a report; otherwise, any additional branch of the phylogenetic tree would deserve another article.

### Authors' response

*Firstly, DEAD Helicase domains belong to the P-loop containing nucleoside triphosphate hydrolases fold, whereas DnaQ-H domains belong to the RNase H fold. It is not possible that a reliable searching with MAEL sequences can retrieve both DnaQ-H domains and DEAD helicase domains. Secondly, since no similarity exists between the MAEL/DnaQ and DEAD domains, it is not reasonable to align them together and infer their evolutionary history. Thirdly, we agree with the reviewer that similar structural fold alone does not provide sufficient evidence of common ancestry *[75]. *However, significant sequence conservation, structural resemblance and catalytic residue conservation may strongly indicate evolutionary relationship *[71,75]. *In our study, the proposed evolutionary relationship is established on the basis of three lines of evidence: 1, sequence similarity via PSI-BLAST, which provides the most straightforward evidence of homology *[75]; *2, similar structural fold; 3, ancestral protist MAEL domains have DnaQ-H characteristic residues. We thank the reviewers for their efforts*.

## Supplementary Material

Additional File 1 **A complete multiple sequence alignment of MAEL domains**. The domain sequences are represented by an abbreviation of species name followed by database ID and domain regions. The consensus in 75% of the sequences is shown below the alignment based on default amino acid classes in Chroma. The numbers in bracket are indicative of the excluded residues from sequences. Species name abbreviations refer to Figure 2 legend. Click here for file

Additional File 2 **Structural alignments between five different DnaQ-H domains showing the plasticity of structural conformations of α5 and α6**. (A) The cartoon structures of four DnaQ-H domains are shown with different colors, in which 2P1J is colored with red. For the structure of 1J53, the NewCartoon diagram is shown with α helices in pink and β sheets in yellow. (B) The structural alignment of 1J53 and 2P1J. The structure of 2P1J is colored in red whereas for 1J53, its α helices are colored in pink and β sheets in yellow. (C) The structural locations of active site residues in both 1J53 and 2P1J domains. Click here for file

Additional File 3 **The conservation of MAEL-specific residues in *E. coli *DnaQ-H domain (1J53:A)**. The cartoon drawing of protein structure is shown. The α helices are shown in pink, β sheets in yellow and loops in cyan. The DnaQ-H specific residues (DEDHD) are highlighted with acidic residues (D and E) in light red and basic His in light blue, whereas three MAEL-specific residues (EHH) are highlighted with acidic Glu in red and basic His in blue. Click here for file

Additional File 4 **Evolutionary relationship between DnaQ-H and MAEL domains**. Click here for file

Additional File 5 **Materials and methods**. Detailed description on materials and methods used in the present study. Click here for file

Additional File 6 **Profile-profile alignment among MAEL, DnaQ (Exonuc X-T, Pfam: PF00929), and DEAD (Pfam: PF00270) domains by the logomat-p program**. Click here for file
