# Supplementary material for: Functional insight into Maelstrom in the germline piRNA pathway: a unique domain homologous to the DnaQ-H 3'–5' exonuclease, its lineage-specific expansion/loss and evolutionarily active site switch
Source: Biol Direct. 2008 Nov 25;3:48. doi: 10.1186/1745-6150-3-48 (PMC2628886; doi:10.1186/1745-6150-3-48)
Supplement: Additional File 1 — A complete multiple sequence alignment of MAEL domains. The domain sequences are represented by an abbreviation of species name followed by database ID and domain regions. The consensus in 75% of the sequences is shown below the alignment based on default amino acid classes in Chroma. The numbers in bracket are indicative of the excluded residues from sequences. Species name abbreviations refer to Figure 2 legend. [file 1745-6150-3-48-S1.pdf]

```
>Am_110759058(71-301)            LNLKREQEFQQKMLQYIDSVVS--MGLLHNTEFAVAQF---SLENG--VENIYHEVLKMKIP----LGWKRDAIETSQQTHQI-PIELE----DGQSD-FSYMFNELTKFLESN--KTGNKFPPLFTAKD---LS
>Ag_118793711(106-335)           KNVLEKQEFYFISMAYFCRTN---TGVHLPAELAVVRY---SLEGG--VKDKLHMFINPGRLP---IGMAYDAQRHAEEDHQL-PLPPNA---MGVSD-YGDVAMRLFSFLLQN-----DDMPLLFTDET---DV
>Aa_108883695(5-235)             PKELEKLEFYFISFAYFCVTS---GGTYIPAEMGLVRY---SLKDG--VMDKLHMFIDPGKLP---LGMAYDAKQHSESDHQL-PIPPDA---KGEKD-NDEIILKLFSFLSQQ-----EKMPPLFTETN---DI
>Aa_108875394(112-344)           NNALEKLEVFFMSCNYFCKTS---TEAFVPAEIALIKY---NLELG--VLDKLHELINPVRLP---LGLAHEALTYSEQTHEL-PTPPNA---MGETD-FYTVLQKILSFTDYN--SKPHKKLAIMTDAK---EV
>Cp_170030346(115-348)           NNALGELEVYFVSCNYFCVSL---SGEYVPAELAIIKY---SLNDG--VMDSLNVLINPTDLP---LGMALDAKTHSSSTHQL-PVPPDA---LGEAN-YEKILRQILKFFKNT--SGSKVVPPIFTWNK---DI
>Cp_170031325(116-346)           SNELEKVEFFFISFAYFCVTS---NGTYIPAEMGLVRY---SLRDG--VKDRLHMFIDPGKLP---LGFSYDAKVHSESDHGL-PIPPDA---MGEKD-NDEIVLRLFNFLSQG-----EKMPPLFTETT---EI
>Cp_170031446(1-207)             --------------------------------MALAKY---SLETG--VMAKTHEYINPQKIP---LGLALDAITWSEETHRL-PVPPDS---KGETD-FSVLLEKLLNFIDFEE-EKKCKNFLLFTDAK---DV
>Nv_156543251(105-307)           -----------------------------------------NLPDN--EKARYKTMAKQTKEQPIKLGFTSMAKDHSSNTHEI-PLNPP----FGKNN-YQEIFKDICKFIEPG--KKNGLLPPVYTMHT----V
>Ll_83933914                     AQKISSKTYFVIHINYFCQSR(4)EPHYDAAELAILDF---SLEDG--IKRGMHTFMKLDTLP---YGYSFEATRHTNDTHQL-PLPPDT---IGQTT-IREAVLEVINFISEV---DDDVCPPIFTFDD---HI
>Gm_78540983                     -----------IAVNYFTKTLK--GNVYIPAELSVCEY---SLKQG--VNRIFHTLINPGTNV---YGHQYEAQHHSETTHNL-PLPPNA---MGDEN-LGTIYNEVLKFLGAT-----DEYPPLYTVRE---NI
>Dp_67840782                     -------------------------------------------------------MINPGHII---YGQSRDAQDHSKTTHKL-PLPPQA---FGETN-MGKLYIDIFNWLSVR(4)LDQDPVIVYTTPE---LM
>Dy_33328997(87-323)             SYDLENAKFVFATFNYFTKALT--TDVYVPAEFAACEY---SLKEG--VRSIYSTMIDPGQII---FGQGSDALHHSSTTHDL-PLPPNA---LGEKN-MAKLYRNIVCYLTKC--QGADKPLIVFTPTE---NI
>Tc_91081127(107-337)            ATDLDEKIFLVIHINHLAYYPT--EDKYFICEIAIAAV---SLKNG--VEDVFHRIVKPGKLP---LGYYGGALTHSKETHQMLELVQDE---PYENN-TREVFNEMTSFLKLW--RGKGSDSIVYADEK---TH
>Dm_21429066(102-338)            SHDLENAKFVFVAFNYFTKALT--TDVYVPAEFAACEY---SLKEG--IRSIYSTMIDPGQII---FGQGSDALLHSSTTHDL-PLPPNA---LGEKN-MTKLYRNIVDYLSKC--QGKGKTLVVFTPAE---NI
>Ci_23575304                     LKGPAGTIFHILSFQSIYELPN--EEGYKPCEVACIKY---SIERG--IIGEWHQFINPGAIE---LGLRAEVQVHTEKTHRI-SEDLYS---QGRDD-YRNIWKELFLFTGSD--VENS----LLCMEN---EI
>Ci_23576040                     EVDVLKEHFFIISFQSLYELPD--EEGYLPCEVTCVDY---TLHGG--IEGIWHNIIDPGAFK---AVLMSEVKFFREGTHQI-GRDCE----YARSN-YYELWKELVAFIRQRS-QCGRTLPPIYCRMS---EV
>Cs1_in_Ensembl(126-353)         NMDVKQEHFIIISFQSLYELPG--EEGYLPCELTCVDY---TLSGG--IQNYWHQIIDPGAYK---PVLMSEVKFFRERTHQI-SRDCG----LARSD-YYAMWRELVAFIRGASRDGDGRIPPIYSRMS---EI
>Cs2_in_Ensembl(527-752)         PEGGASTVYHIISFQSIYELPN--EEGYKPCEVACVKY---SLKRG--VIGQWHRFINPGKIE---LGLRAEIKCYSETYHRI-SEEMYK---QFCSR-YTAMWMELLSFVEDE--ADSESLPPLLCMEN---EI
>Sp_115675725(110-291)           GQDITHHRFYMIAVKTLCQLP---DDTYLPCELACVEY---TMSAG--ITKRFHCFP-------------------------I-PAEWFD---QYNHN-YKEIWTDLIAFINPQ---RQHVMPALFCELR---CR
>Gg_118083700(104-329)           DQAVLADTFYFLNVYSHGKLPSHCDQRFLPCEIGCVKY---SLQEG--IMADFHHFIDSEVPP---RGYRYHCQAASDATHKI-PISGFH---LSRTC-YPVVIRELLQFAQPA----RGAWPRFYCKSD---DR
>Xt_118404620(121-346)           NKAPDQHVCYFINIFSHGDMPSLCEQRYVPCEIACVRY---SLREG--ILGSFHDFIDPGELP---RGFRYHCQSGSASTHQI-PISGFE---LANSD-YHNMFRKLCSFVCPT----PCPVVPVYTKAN---DI
>Md_126306192(110-334)           DQAVLGSFFYFLNIFSHGELPPHCEQRFLPCEIGCIKY---SLQEG--IVAEFHRFIDPGEVP---RGFRFHCQAASDASHKI-PISNFH---SGDD--YAVVLQNLYRFISPN----QSSWPPVYCKSD---DR
>Ec_149707838(107-331)           DQALLGGIFYFLNIFSHGELPPHCEQRFLPCEIGCVKY---SLQEG--IIADFHSFINPGEIP---RGFRFHCQTASDSSHKI-PISNFE---PGRD--QAVVLQNLYRFIHPS----PGSWPPIYCKSD---DR
>Bt_84000365(107-317)            DQALLGGIFYFLNIFSHGELPPHCEQRFLPCEIGCVKY---SLQEG--IMADFHSFINPG-----------------DSSHKI-PISHFE---SGHD--QATVIENLYRFIHPN----PGNWPPIYCKSD---DR
>Mu_109019354(107-331)           DQALLGGIFYFLNIFSHGELPPHCEQRFLPCEIGCVKY---SLQEG--IMADFHSFINPGEIP---RGFRFHCQAASDSSHKI-PISNFE---RGHN--QATVLQNLYRFIHPN----PGNWPPIYCKSD---DR
>Rn_109498972(107-331)           DQALLGGVFYFLNIFSHGELPPHCEQRFLPCEIGCVKY---SLQEG--IMADFHSFINPGEIP---RGFRFHCQAASDSSHKI-PISNFE---FGHD--QATVLQNLYKFIHPN----PGNWPPIYCKSD---DR
>Hs_20306906(107-331)            DQALLGGIFYFLNIFSHGELPPHCEQRFLPCEIGCVKY---SLQEG--IMADFHSFINPGEIP---RGFRFHCQAASDSSHKI-PISNFE---RGHN--QATVLQNLYRFIHPN----PGNWPPIYCKSD---DR
>Mm_29748051(107-331)            DQALLGGIFYFLNIFSHGELPPHCEQRFLPCEIGCVKY---SLQEG--IMADFHSFIHPGEIP---RGFRFHCQAASDSSHKI-PISNFE---FGHD--QATVLQNLYKFIHPN----PGNWPPIYCKSD---DR
>Cf_73960729(107-331)            DQALLGGIFYFLNIFSHGELPPHCEQRFLPCEIGCVKY---SLQEG--IMAEFHRFINPGEIP---RGFRFHCQAASDSSHKI-PISNFE---SGYD--QATVLQNLYRFIHPN----PGNWPLIYCKSD---DR
>Ss_147901147(107-331)           DQALLGGIFYFLNIFSHGELPPHCEQRFLPCEIGCVKY---SLQEG--IMADFHSFINPGEIP---RGFRFHCQAASDSSHKI-PISHFE---SGHD--QATVLQNLYRFIRPN----SGKWPPIYCKSD---DR
>Ce_3873737(525-803)             IDGIRDLRFLLASVQTYGNI----DGECMLAEMAMNEF---SLFSG--IVEKFHAIVGPWMPE--SESHRRRASRHALETHRI-PLQNN----FATIT-KKRLVEEILGRVEPS(39)NDRRFILVLQSE---LD
>Cb_39591559(528-806)            IDGIRDLRFLLASVQTYGNI----DGECMMAELGLNEF---SLFSG--IIEKYHAIVGPWQTD--NESQRRRASRHAFETHRI-PLQHS----IATVS-KRKLVEEILGRVEPS(39)NDRRFILVLQSE---LD
>Bm_170586486(160-295)           LDEIRRARFLFISVQTYGNV----DGICIPAEIAICEF---NLKHG--IIDKYTSIIGPWRLN--NEIQRRRAEFHANETHQI-HLDMFG(6)PTQNS-VLLFYFILFRIAS-----------------------
>Eh_67477376(315-532)            EINIFEFPIHFFDFEFSSSK----TDGIIPLELGISTY---YLKENK-EINFYHTLIKPS--------HYNTSFERAVGVHGI-DLHMN-----YTQS-YSEIIEGLTNYLNSF-----KGKKLLVLKDE---TI
>Eh_67476664(42-258)             AQTLENGVFHFYDFEYAAQF----SEKIFPIEIGISSY---SLKENK-EIASYHKLLYPG--------KFKNVFARTQMIHGI-DARDP----RLEQN-YSLVCIELIKYIEQF-----PGLAFFVSKEE---SL
>Eh_67466465(15-246)             GPRVEQQLFFILDFQISYKT----DICTVPVEICIKP----TLLNGTINIECFQTIINQPIP----IQHFLNSKHYTDFEHGI-SQENNP---VPQTD-FDFLWKKINTFIKSN(5)DSSMLPIIICTP-----F
>Eh_67484628(16-237)             GEQAIKQPFFFIDFGINYTT----DETCVPIEICIQPF---TLDPNSAPVKPFLQIINEVVP----NQYLLRAKQHADFEHGI-TPENNP---SHPTD-FLLLWNTINNFIWTF(4)GETFVPIVICLS-----F
>Eh_67477376(12-227)             EIITNDIPVHFVNFEYCARA----MKMTYPIELGICTY---KMSEFK-LLGEFHQLIYCD------ISEFINKKQL-TNHHGL-DSKSQ----FLRKD-YKNIVNELMKYLKSF----SNNIYCIVKRKE---IR
>Ed_167389979(315-532)           EINIFEFPIHFFDFEFSSSK----TDGVIPLELGISTY---YLNESK-EINFYHTLIKPS--------HYNSSFERAVGVHGI-DLHMN-----YTQS-YSEIINGLTNYLNSF-----KGKKLLVLKDE---TI
>Ed_167384018(42-258)            TQTLENGVFHFYDFEYAAQF----SEKIFPIEIGISSY---SLKENK-EINSYHKLLYPG--------KFKNVFARTQMIHGI-DARDP----RLEQN-YSLVCIELIKYIEQF-----PGLAFFVSKEE---SL
>Ed_167391142(15-246)            GPRVEQQLFFILDFQISYKT----DICTVPVEICIKP----TLLNGTINIECFQTIINQPIP----IQHFLNSKHYTDFEHGI-SQENNP---VPQTD-FDFLWKKINSFIKSN(5)DSSMLPIVICTP-----F
>Ed_167395021(16-237)            GEQAIKQPFFFIDFGINYTT----DETCVPIEICIQPF---TLDPSAEPVKPFLQIINEVVP----SQYLLRAKQHADFEHGI-TPENNP---SDPTN-FLLLWNKINNFIWTF(4)GEAFVPIVICLS-----F
>Ed_167389979(12-227)            DIIKNDIPVHFVNFEYYART----MKITYPIELGICTY---KMSEFK-LLGEFHQLIYCD------INEFVDKKKI-KNHHGL-DTTSS----FLRND-YKNIVNELTKYLKSF----NDKICCIVKRKE---TR
>Tv_tviv441h03_q1k_3(253_468)    IGGVGNAIFVSFDAEAVLVSY---GSIPLPLEIALVP----VVKNE--KLPPFHCFLHPGHVE-DCVTAIKLSCGLVDGAHCI-PFKCAS---FLRRD-YAAVAGEINRFLSC--------KQVVLVNKG----T
>Tb_71748114(363-570)            MGGMQNAIFVFLDVEAAVVQK---NSVPLPLEIALVP----SGGDH--SFQPFHCFLHPGVIE-NSQVALGLSCGAIPSSHHV-PLNNVT---FLRRD-YTKVAEEISQFLSC--------ERVVLINKG----S
>Tr_congo1317f04_p1k_5(268_485)  RGGLGKAVFVCFDIEAAIVHR---NCVPQPLEIALVP----CGSNH--SLTHYHCFIHPGKVV-DNETALKLSCGFVPGSHYI-PFQGAS---FLRRD-YANIANDLRSFLSY--------ECVVFVNKD----S
>Lb_LbrM35_V2_6220(281_523)      AGDWSNITFVAVDTEAYAVME---HSVPLPAEYAFLPI(5)STSSA--VLSPLHFFCHPGNVE---AENEENVLYNCLNTHLI-PYHSAT---FLTDNFYDKAVLVDRQFVRN--------PSVILISKG(4)PT
Consensus/75%                    ...h....abbbsh..hhph....p..hhPhElth.pa...*Lppt...bs.aHpblpPs........b..psb.hsp.sH.l.sb.s......hpps.b..lhpplhpalp...........lbs.pp.....
```

```
>Am_110759058(71-301)              PVV---ESLLIKMIDASNGSI----DDFIIYSIEALFGA-LRNAAVQ(4)RSIPLIVAENEFSKDFLCN----TRGLECDFHKILDI---SQYCSKSIVKRWAFTICDY---CCEYLNIKLIEGVHRPKETPFLQ
>Ag_118793711(106-335)             PRV---ESMLEHILSDHLSEI-----ELRICPLAELFFR-LKQNVEL(6)TFPSVYIAQQIITKDVYDY----TKGISCDYHEEKDN---VLYCPLSRCIRWAYIISDN---CCQDMGIEPIPGKHVPLNANTNP
>Aa_108883695(5-235)               RMV---ENILKGILNQGSMDE----NTLLVCPLSELFYQ-LKRATES(6)TFPSVHIAQAIIQKDVYEY----TKDISCEFHEDQGN---GKYCPLSRCVRWAYIISDS---CCLDLSIEMKPGRHLPMNADTAL
>Aa_108875394(112-344)             PVI---ESLLSQLNDDVKLEY-----QFLVIPLGEFFFH-LKRATEK(6)TFPTKTVADILLKKDAYEY----TSGIACDFHEKLGN---QRFCALSKVVRWSYIISDN---CCLDLSIDLIAGRHLPSNADTTL
>Cp_170030346(115-348)             PMV---DSILRGILEATDLDY----VKFSILPLIDFFYN-LKLATED(6)TFPSIHLAKALLEKDVYAY----TAGIACDVHEQLNN---QVACALSRVVRWAYVISDS---CCLDVGIEMEKGRHLPHNMTTLS
>Cp_170031325(116-346)             KMV---ENILKGILTQANMDE----NTLLVCPLSELFYQ-LKRATES(6)TFPSVHIAQAIIQKDVYEY----SKDISCDFHEEKGN---GKYCPLSKCVRWAFIISDS---CCLDLSIEMKPGRHLPLNADTDL
>Cp_170031446(1-207)               GET---ENILSQFTEDSKILF-----PFLVCPLGEFFYQ-LKVATER(8)NFPGRTIADVLLKKDPYEY----TGGISCDFHEDLGN---PRYCALSRVIRWAYMISDN---CCLDLNIEVISGQHLPANADTTL
>Nv_156543251(105-307)             EDV(4)KSVLSRLAAADQKPD----EYLRLYSFEDLFAT-ICNAMFE(4)VKIEPLVAKIELKRDPFAY----KLGLECQFHSDLKA--GVLYCSQSYTKRWIFVLCHH---CCKRLGITMIPGIHMPIENPISP
>Ll_83933914                       PVV---RSIFDDVLSQLATAN---PGDVRIYSLSELLFH-LKAATHN(9)PFASVFLAESYLERGTFDY----AEGLPCEYHQEIDR---VPNCSQTKIKGWAFSIIRS---CAPDLGITFMEGEHEPMNTQPTP
>Gm_78540983                       HIV---VSVLDFLKSDIRASN----ITLNVYPIQYLFYV-MKESTCE(5)KPKSFHITDAHFERDCFEY----QNGIACQFHEDKDK---SKYCTQSIVTRWGYMFSDY---MCRDIAVPLIAGRHIPQNTNLEA
>Dp_67840782                       PVV---KSCFRYLASEAEIDED--ERKIMVFDIYHLFYT-LKKSVLD(6)DRINFHVTNNFFVKDFFEY----TEGIACDYHEKIDR---SKYCTNSMVKRWGFTFSDY---MCADLAIPLQPGKHIPLKVKPNY
>Dy_33328997(87-323)               AMV---NSCFRYLECEDDSGDG--GRKIQVFDIQYLLFI-LKKAVMD(6)EKINKFVTDAFFKKDFFEF----TSGIACQYHEDNDR---TKYCTQSMVTRWAYTFSDF---MCGDLAITVQPGKHIPAETKPNY
>Tc_91081127(107-337)              EMI---TKVIDNFCQEFNYPD-----EIKVYNFQYLFFA-LRNSVAA-RTVWPTETYSSTELEKDLYSY----TPDISCEFHEMSDI---SVYCSKSIVTRYCYTLCDH---CCTDLNIQLVAGFHVPKNSRIAV
>Dm_21429066(102-338)              TMV---KSCFRYLECDDDFRDG--GEKIQVFDIQYLLFI-LKKEVMN(6)EKINKFATDAFFKKDFFEF----TAGIACQYHEDNDR---TKYCTQSMVTRWAYTFTDF---MCGDLAITVQPGKHIPAQTKPNY
>Ci_23575304                       RKN---CFCLHWLAEK-AYNN-----HFEVYGFESITSA-LYGYTEP---VCLSPVLITAGCNSSMFDY----ELGIKCEYHEEIE----CTCCTLLTVKRCCYWMTNT---FSGIYNVDITES-HLPVKSQSSF
>Ci_23576040                       RKN---QFCLNWLSQNAQMAN----QLSKLHELEHLARE-LICNAVN---TKFALTSIEDGFNSSMWEY----EPGIKCRYHEDVD----CYFCTMLTIKKCCFWMSEV---LAPYYGFELTRN-HLPEESDEPL
>Cs1_in_Ensembl(126-353)           KKN---VFCLDWLAQNAGMAN----QLCKVFELEFLVSE-LISNAMQ---TKTPLSSIESGFTSSMWEY----EIGIKCEYHEEID----CYFCTMLTIKKCCFWISEV---LSPIYHFPLTEN-HLPSEQADCF
>Cs2_in_Ensembl(527-752)           RKN---SFCLQWLANKGGYPN-----LFRVYGFESMASA-LYANAQR---TPLALSLIQSGCNSSMFDY----EAGIKCYFHEENE----CTCCALLTVKRCAYWMSDA---FLGVYDYDITES-HFPVKSEASF
>Sp_115675725(110-291)             EEV---EYTLEWLARNAGMSH----SLRKVYETELLILE-LFRNAGE---IVPSRGQVSSLLASSAWDY----IWD-------------------------TSYCLSDA---VCARYNVELTPN-HIPEAETPDA
>Gg_118083700(104-329)             FRI---SWCLERMASIAGVDS-----PLELLTVEDLVIK-LYQKKYH---KEPSKTWVSRELDVVLWDF----SSNTRCEWHEEND----ILCCALASCKKIAYCISKS---LAGVYGVSLTAA-HLPPKDCVSN
>Xt_118404620(121-346)             YRV---DWCLQWLANKAGMEN-----HFRVQEVETLIIK-FYQDKLQ---EEPSRPTVSRLLDVVQWDY----SSNTRCKWHEDND----MWCCALASCKKIAYCISKA---LASVYGVTLTPA-HLPNPERSRN
>Md_126306192(110-334)             YRV---NWCLKHMAKKLETRQ-----ELELLTVEDLVVG-IYQQKLH---KEPSKTWVRSLLDVSMWDY----SSNTRCKWHEKND----ILFCALAVCKKIAYCISNS---LGTLFGIPLTEA-HVPLQDYEAS
>Ec_149707838(107-331)             AKV---NWCLKHMAKASEIRQ-----DLELLTVEDLVVG-IYQQKFL---KEPSKTWVRSLLDVAMWDY----SSNTRCKWHEEND----ILFCALAVCKKIAYCISNS---LATLFGIQLTVA-HVPLQDYEAR
>Bt_84000365(107-317)              ARV---NWCLKHMAKSSEIRQ-----DLELLTVEDLVVG-IYQQKFL---KEPSKTWVRSLLEVAMWDY----SSNTRCKWHEEND----ILFCALAVCKKIANCISNS---LATLFGIQLTEA-HVPLQDYEAS
>Mu_109019354(107-331)             TRV---NWCLKHMAKASEIRQ-----DLQLLTVEDLVVG-IYQQKFL---KEPSKTWIRSLLDVAMWDY----SSNTRCKWHEEND----ILFCALAVCKKIAYCISNS---LATLFGIQLTEA-HVPLQDYEAS
>Rn_109498972(107-331)             ARV---NWCLKRMERASEIRQ-----DLELLTVEDLVVG-IYQQKFL---KEPSKTWVRSLLDVAMWDY----SSNTRCKWHEEND----ILFCALAVCKKIAYCISNS---LATLFGIQLTGA-HVPLQDYEAS
>Hs_20306906(107-331)              TRV---NWCLKHMAKASEIRQ-----DLQLLTVEDLVVG-IYQQKFL---KEPSKTWIRSLLDVAMWDY----SSNTRCKWHEEND----ILFCALAVCKKIAYCISNS---LATLFGIQLTEA-HVPLQDYEAS
>Mm_29748051(107-331)              ARV---NWCLKRMERASEIRQ-----DLELLTVEDLVVG-IYQQKFL---KEPSKTWVRSLLDVAMWDY----SSNTRCKWHEEND----ILFCALAVCKKIAYCISNS---LATLFGIQLTGA-HVPLQDYEAS
>Cf_73960729(107-331)              ARV---NWCLKHMAKASEIRQ-----DLELLTVEDLVVG-IYQQKFL---KEPSKTWVRSLLDVAMWDY----SSNTRCKWHEEND----ILFCALAVCKKIAYCISNS---LATLFGIQLTDT-HVPLQDYEAS
>Ss_147901147(107-331)             ARV---NWCLKYMAKSSEITQ-----DLELVTVEDLVVG-IYQQKFL---KEPSKTWVRSLLDVAMWDY----SSNTRCKWHEEND----ILFCALAVCRKIAYCISNS---LATLLGIQLTEA-HVPLQDYETS
>Ce_3873737(525-803)               LMV---DS-MKHLANNVGFHY(4)VTPNCFVIVEAFVEA-ISDIMNE----KIDVETMRWFSLLGQKVD(10)GTDFHCARHSEPK----SNFCASVTVGRTCCIVYHVIGSFFRRYHLKKIPTAHQPSSSNSVQ
>Cb_39591559(528-806)              LMV---ES-MKHLAKTVGFNY(4)VHQNNFVIVEAFVEA-ISDIMGE----KVDMETMRWFSLLGQKVD(10)GTDFHCSRHSEPK----SNFCAAVTVGRTCCIIYHVLGSFFRRYHLKKIPTAHQNSSSFNSM
>Bm_170586486(160-295)             -TM---CNCLKEILGRCEPTI(4)GVFVGLYRDGIAEFD-----------------------------------------------------------------------------------------------------
>Eh_67477376(315-532)              GGD---VLCFNNLFGYIQQSIP---NDYIFTTHHFLLEY-IATMKGFDNTKAKECGILMNDVYKQLKI-------CDKCTYHQLLAD---RYHCGLQDARHTALA-ILI---ILKDIGYEIKNNDKFPFCVVHKN
>Eh_67476664(42-258)               AGD---KKCIDEIFLRGNVPIP---KQIRFITHIQLFDY-WCSIQHIELHEKSSFILNHIFKQLECA---------ERCEYHKKINQ---KYHCALSDARHTSLM-ELI---CMKSYGATIIGSDTLPSVKFVKS
>Eh_67466465(15-246)               ISS---VQCVEFLASQAKVSDVRRSIFNTMFSVDDFVEC-VNRFKE----IIPNTNAIYNFY-KPLVCWTC--NNDFKCDFHKSNGT--RTFCCSKTNSEYLASTLCDL---YKTIKSKIFVAS--MPSQVQSMV
>Eh_67484628(16-237)               RRG---VQCIENLATKAGLYD(4)SGFMHALPFEAFVKF-FYESQG----KVVPISAIPKCL-CPLLSYIP--ETKYKCSFHSNIKT--RSFCCCQSNVAYLVG--------------ELTCNF-NPYNQFNIL
>Eh_67477376(12-227)               NPS---YECFVKLFQLANEQF----KNFKFIKESEFLYL-LC-SFYN-KSIENPFSLLMSVS-TRIGT-------IEKCQIHKEIM----SKGCALDSVKLSATSIFV----IMKNLKISFKEIDQLPILVFINK
>Ed_167389979(315-532)             GGD---VLCFNNLFGYIQQSIP---NDYIFTTHHFLLEY-IATMKGFDNNKAKEYGILMNDVYKQLKI-------SNRCPYHQLLAD---RYHCGLQDARHTALA-TLL---ILRDIGYKIKNNDKLPFCVIHKN
>Ed_167384018(42-258)              AGD---KKCIDEIFLRGNVPIP---KQIRFITHIQLFDY-WCSIQHIELHEKPSFILNHIFKQLECA---------ERCEYHKKINQ---KYHCALSDARHTSLM-ELI---CMKSYGATIIGSETLPSVRFVKS
>Ed_167391142(15-246)              ISS---VQCIEFLASQAKVSDVRRSVFNTMFSVDDFVEC-VNRFKE----IIPNTNAIYNFY-KPLVCWTC--NNNFKCDFHKSNGT--RTFCCSKTNSEYLASTLCDL---YKTIKSKIFVAS--VPPQVQSMV
>Ed_167395021(16-237)              RRG---VQCIENLATKAGLYD(4)SGFMHALAFEEFVKF-FYESQG----RVVPISAIPKCL-CPLLSYIP--ETKYKCSFHANIKT--RSFCCCQSNVAYLVG--------------ELTCTF-NPYNQSNIL
>Ed_167389979(12-227)              NPS---YECFVKLFQLANEQF----KNFEFIKESEFLYL-LC-SFYN-KSIESPYSLLMSVS-TRIGS-------IEKCQIHKEIM----SKGCALDSVKLSANSIFV----IMKNMKIPFKEIDQLPILLFVNK
>Tv_tviv441h03_q1k_3(253_468)      LMD(4)RWVFAAARALENEGD(8)LLSMPCYSFDVVWEF-FSD-------------EDVKKSYDNLKS-----LPKKPCSYHRKIS(11)SAHCALEDAETLCDVLRPL---IQRV------------------
>Tb_71748114(363-570)              LMD(4)RWVFGAARIAESSNM(4)LEDIACFDIQALKKL-LAQ-------------GEWRETKGDAAE-----THGEFCWYHAGMQ(11)ESHCALKDAQVIHGVVQRY---L---------------------
>Tr_congo1317f04_p1k_5(268_485)    LMD(4)RWVFAAACEVGNAEM(4)LEDIPCFDIETVKEL-LVG--------------SGSSTRENITKT----NFEAPCWYHDKMH(11)GCHCALKDAMNLRNEIEKY---LKPLQQQATD------------
>Lb_LbrM35_V2_6220(281_523)        LMD(4)RWLYAAAALQWHNGH(20)EDIYCFDISVLEAVALER-------------GGIADSQGTPSLT(9)-EENGYCWYHSVVN(6)GDVHCAMHDAYTLAGRIKAV---LPA--------------------
Consensus/75%                      ..s...p.sbp.bhp.s.h........bbhbshp.bh.h.l.p..........s..hh..hh..shbp.......sh.CpaHpp........hCsb..s.phshhbpp....hh..b.h.b..s.phP.p.....
```
