# Supplementary figures and images for: Functional insight into Maelstrom in the germline piRNA pathway: a unique domain homologous to the DnaQ-H 3'–5' exonuclease, its lineage-specific expansion/loss and evolutionarily active site switch
Source: Biol Direct. 2008 Nov 25;3:48. doi: 10.1186/1745-6150-3-48 (PMC2628886; doi:10.1186/1745-6150-3-48)

## Slide 1
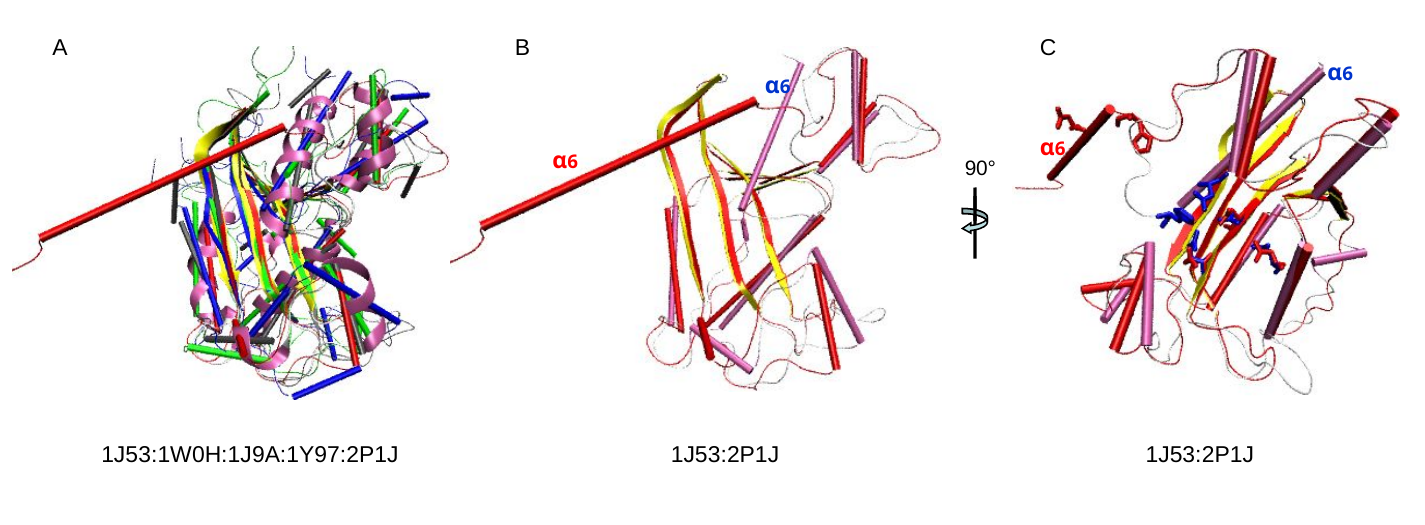

A
B
C
α6
α6
α6
α6
90°
1J53:1W0H:1J9A:1Y97:2P1J
1J53:2P1J
1J53:2P1J

Supplement: Additional File 2 — Structural alignments between five different DnaQ-H domains showing the plasticity of structural conformations of α5 and α6. (A) The cartoon structures of four DnaQ-H domains are shown with different colors, in which 2P1J is colored with red. For the structure of 1J53, the NewCartoon diagram is shown with α helices in pink and β sheets in yellow. (B) The structural alignment of 1J53 and 2P1J. The structure of 2P1J is colored in red whereas for 1J53, its α helices are colored in pink and β sheets in yellow. (C) The structural locations of active site residues in both 1J53 and 2P1J domains. [file 1745-6150-3-48-S2.ppt]

## Slide 1
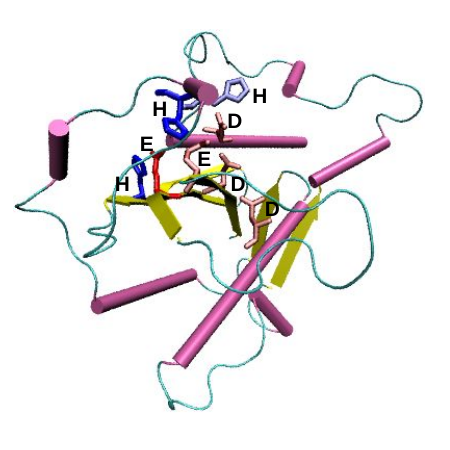

H
H
D
E
E
H
D
D

Supplement: Additional File 3 — The conservation of MAEL-specific residues in E. coli DnaQ-H domain (1J53:A). The cartoon drawing of protein structure is shown. The α helices are shown in pink, β sheets in yellow and loops in cyan. The DnaQ-H specific residues (DEDHD) are highlighted with acidic residues (D and E) in light red and basic His in light blue, whereas three MAEL-specific residues (EHH) are highlighted with acidic Glu in red and basic His in blue. [file 1745-6150-3-48-S3.ppt]

## Slide 1
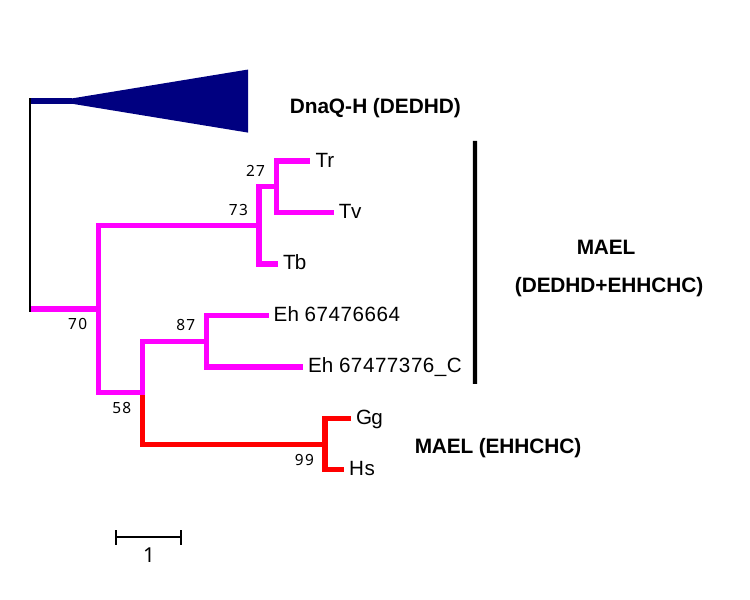

DnaQ-H (DEDHD)
MAEL
 (DEDHD+EHHCHC)
MAEL (EHHCHC)

Supplement: Additional File 4 — Evolutionary relationship between DnaQ-H and MAEL domains. [file 1745-6150-3-48-S4.ppt]
