# Supplementary material for: Functional insight into Maelstrom in the germline piRNA pathway: a unique domain homologous to the DnaQ-H 3'–5' exonuclease, its lineage-specific expansion/loss and evolutionarily active site switch
Source: Biol Direct. 2008 Nov 25;3:48. doi: 10.1186/1745-6150-3-48 (PMC2628886; doi:10.1186/1745-6150-3-48)
Supplement: Additional File 5 — Materials and methods. Detailed description on materials and methods used in the present study. [file 1745-6150-3-48-S5.pdf]

Materials and Methods

Database searching

The C-terminal sequences of MAEL from several species were used as queries for PSI-BLAST searches [1] against the protein non-redundant (NR) database at National Center for Biotechnology Information (NCBI) with a profile inclusion expectation (*E*) value threshold of 0.005. A substitution matrix of BLOSUM62 and the gap penalty (existence: 11 and extension: 1) were utilized for scoring. The searches were iterated until convergence. Other homologous sequences were also identified through BLAST searching in Ensembl database [2] and TBLASTN searching in NCBI translated database with default parameters. Furthermore, for other protist homologues, we searched the database of the GeneDB project [3]. A total of 47 homologous sequences have been collected in these searches by May 1, 2008. Some more homologous sequences can be retrieved from NCBI database (July, 2008) when we prepare this manuscript. However they are not included in the current analysis since new sequences do not influence our result.

Sequence analysis

In order to construct the multiple alignment of MAEL domain sequences, we first utilized the Muscle [4] and Promals [5] programs. The logomat-p program [6] was also used to align sequence profiles of vertebrates, insects, nematodes, sea squirts and protists. Careful manual adjustments were conducted to avoid introducing gaps into the sequences where consensus secondary structures are occupied. The final alignment is colored using Chroma [7]. The putative secondary structures for most MAEL domains were predicted by PSIPred program [8]. A consensus-deriving secondary structure prediction program, SYMPred, was also utilized for some specific predictions (http://zeus.cs.vu.nl/programs/sympredwww/). In the SYMPred prediction, PSIPred [8], SSPro [9], YASPIN [10], and PROFsec (Rost, unpublished) programs were considered and dynamic programming was used as a consensus deriving scheme. Domain composition was deduced by searching protein domain databases Pfam [11] and SMART [12].

Phylogenetic inference

Based on the final multiple sequence alignment of MAEL domains (additional file 1), an unrooted phylogenetic tree was constructed with maximum likelihood (ML) analysis implemented in PhyML program [13] and Bayesian analysis implemented in MrBayes 2.1 program [14]. The ML tree was determined under a Jones-Taylor-Thornton (JTT) model for amino acids substitution with a discrete gamma distribution (four categories), a proportion of invariant and an initial BIONJ tree. A bootstrap analysis with 100 repetitions was performed to assess the significance of phylogenetic grouping. For

Bayesian phylogenetic inference, firstly we used ProtTest 1.3 [15] to determine the best fitting model of amino acid substitution for the data under the maximum likelihood assumption. A WAG model with a gamma distribution (four rate categories), a proportion of invariable sites, and observed amino acid frequencies (WAG+G4+I+F) turned out to be the best model and was utilized in Bayesian analysis subsequently. The Metropolis-coupled Markov chain Monte Carlo (MCMCMC) sampling approach was used to calculate posterior probabilities. Four Markov chains were run 10,000,000 times. The chain was sampled every 100th generation, and burn-in values were determined from the likelihood values. The final unrooted tree diagram was generated using MEGA Tree Explorer [16].

Fold recognition and structure modeling

Protein fold assignment was conducted using *meta* Server (http://meta.bioinfo.pl/), which assembles different state-of-the-art fold recognition programs including meta-BASIC [17], ORFeus-2 [18], and FFAS03 [19] and further evaluates the modeled three-dimensional structures based on a consensus scoring computed by a 3D-JURY system [20]. The domain and structural fold annotation was then assigned for all the candidate hits by checking Pfam domain database [11] and the Structural Classification of Proteins (SCOP) database [21]. Structure-based multiple sequence alignment was built using CE-MC server [22]. The final sequence and secondary structure alignment of MAEL domains with several DnaQ-H domains was established carefully by hand on the basis of CE-MC results, alignment in fold recognition, published literature information, and predicted secondary structures. The final alignment was then used for structural homology modeling, which was performed via Modeller9v1 program based on multiple templates [23]. Structural alignment was conducted by MultiProt server [24]. Non-homologous regions were predicted by Loopy [25]. Disulfide bond prediction was conducted by an artificial neural network method [26]. Structural visualization and manipulations were performed using VMD program [27].

References

1. Altschul SF, Madden TL, Schaffer AA, Zhang J, Zhang Z, Miller W, Lipman DJ: **Gapped BLAST and PSI-BLAST: a new generation of protein database search programs**. *Nucleic Acids Res* 1997, **25**(17):3389-3402.
2. Flicek P, Aken BL, Beal K, Ballester B, Caccamo M, Chen Y, Clarke L, Coates G, Cunningham F, Cutts T *et al*: **Ensembl 2008**. *Nucleic Acids Res* 2008, **36**(Database issue):D707-714.
3. Hertz-Fowler C, Peacock CS, Wood V, Aslett M, Kerhornou A, Mooney P, Tivey A, Berriman M, Hall N, Rutherford K *et al*: **GeneDB: a resource for prokaryotic and eukaryotic organisms**. *Nucleic Acids Res* 2004, **32**(Database issue):D339-343.

4. Edgar RC: **MUSCLE: a multiple sequence alignment method with reduced time and space complexity**. *BMC Bioinformatics* 2004, **5**:113.
5. Pei J, Kim BH, Tang M, Grishin NV: **PROMALS web server for accurate multiple protein sequence alignments**. *Nucleic Acids Res* 2007, **35**(Web Server issue):W649-652.
6. Schuster-Bockler B, Bateman A: **Visualizing profile-profile alignment: pairwise HMM logos**. *Bioinformatics* 2005, **21**(12):2912-2913.
7. Goodstadt L, Ponting CP: **CHROMA: consensus-based colouring of multiple alignments for publication**. *Bioinformatics* 2001, **17**(9):845-846.
8. McGuffin LJ, Bryson K, Jones DT: **The PSIPRED protein structure prediction server**. *Bioinformatics* 2000, **16**(4):404-405.
9. Pollastri G, Przybylski D, Rost B, Baldi P: **Improving the prediction of protein secondary structure in three and eight classes using recurrent neural networks and profiles**. *Proteins* 2002, **47**(2):228-235.
10. Lin K, Simossis VA, Taylor WR, Heringa J: **A simple and fast secondary structure prediction method using hidden neural networks**. *Bioinformatics* 2005, **21**(2):152-159.
11. Finn RD, Tate J, Mistry J, Coggill PC, Sammut SJ, Hotz HR, Ceric G, Forslund K, Eddy SR, Sonnhammer EL *et al*: **The Pfam protein families database**. *Nucleic Acids Res* 2008, **36**(Database issue):D281-288.
12. Letunic I, Copley RR, Pils B, Pinkert S, Schultz J, Bork P: **SMART 5: domains in the context of genomes and networks**. *Nucleic Acids Res* 2006, **34**(Database issue):D257-260.
13. Guindon S, Gascuel O: **A simple, fast, and accurate algorithm to estimate large phylogenies by maximum likelihood**. *Syst Biol* 2003, **52**(5):696-704.
14. Huelsenbeck JP, Ronquist F: **MRBAYES: Bayesian inference of phylogenetic trees**. *Bioinformatics* 2001, **17**(8):754-755.
15. Abascal F, Zardoya R, Posada D: **ProtTest: selection of best-fit models of protein evolution**. *Bioinformatics* 2005, **21**(9):2104-2105.
16. Kumar S, Nei M, Dudley J, Tamura K: **MEGA: a biologist-centric software for evolutionary analysis of DNA and protein sequences**. *Brief Bioinform* 2008, **9**(4):299-306.
17. Ginalski K, von Grotthuss M, Grishin NV, Rychlewski L: **Detecting distant homology with Meta-BASIC**. *Nucleic Acids Res* 2004, **32**(Web Server issue):W576-581.
18. Ginalski K, Pas J, Wyrwicz LS, von Grotthuss M, Bujnicki JM, Rychlewski L: **ORFeus: Detection of distant homology using sequence profiles and predicted secondary structure**. *Nucleic Acids Res* 2003, **31**(13):3804-3807.
19. Jaroszewski L, Rychlewski L, Li Z, Li W, Godzik A: **FFAS03: a server for profile--profile sequence alignments**. *Nucleic Acids Res* 2005, **33**(Web Server issue):W284-288.
20. Ginalski K, Elofsson A, Fischer D, Rychlewski L: **3D-Jury: a simple approach to improve protein structure predictions**. *Bioinformatics* 2003, **19**(8):1015-1018.
21. Andreeva A, Howorth D, Chandonia JM, Brenner SE, Hubbard TJ, Chothia C, Murzin AG: **Data growth and its impact on the SCOP database: new developments**. *Nucleic Acids Res* 2008, **36**(Database issue):D419-425.
22. Guda C, Lu S, Scheeff ED, Bourne PE, Shindyalov IN: **CE-MC: a multiple protein structure alignment server**. *Nucleic Acids Res* 2004, **32**(Web Server issue):W100-103.
23. Marti-Renom MA, Stuart AC, Fiser A, Sanchez R, Melo F, Sali A: **Comparative protein structure modeling of genes and genomes**. *Annu Rev Biophys Biomol Struct* 2000, **29**:291-325.

24. Shatsky M, Nussinov R, Wolfson HJ: **A method for simultaneous alignment of multiple protein structures**. *Proteins* 2004, **56**(1):143-156.
25. Xiang Z, Soto CS, Honig B: **Evaluating conformational free energies: the colony energy and its application to the problem of loop prediction**. *Proc Natl Acad Sci U S A* 2002, **99**(11):7432-7437.
26. Ferre F, Clote P: **DiANNA 1.1: an extension of the DiANNA web server for ternary cysteine classification**. *Nucleic Acids Res* 2006, **34**(Web Server issue):W182-185.
27. Humphrey W, Dalke A, Schulten K: **VMD: visual molecular dynamics**. *J Mol Graph* 1996, **14**(1):33-38, 27-38.
