# Supplementary material for: Functional insight into Maelstrom in the germline piRNA pathway: a unique domain homologous to the DnaQ-H 3'–5' exonuclease, its lineage-specific expansion/loss and evolutionarily active site switch
Source: Biol Direct. 2008 Nov 25;3:48. doi: 10.1186/1745-6150-3-48 (PMC2628886; doi:10.1186/1745-6150-3-48)

A: Profile-profile alignment between MAEL (above) and DnaQ (Exonuc X-T, Pfam: PF00929) (bottom) domains by the logomat-p program.


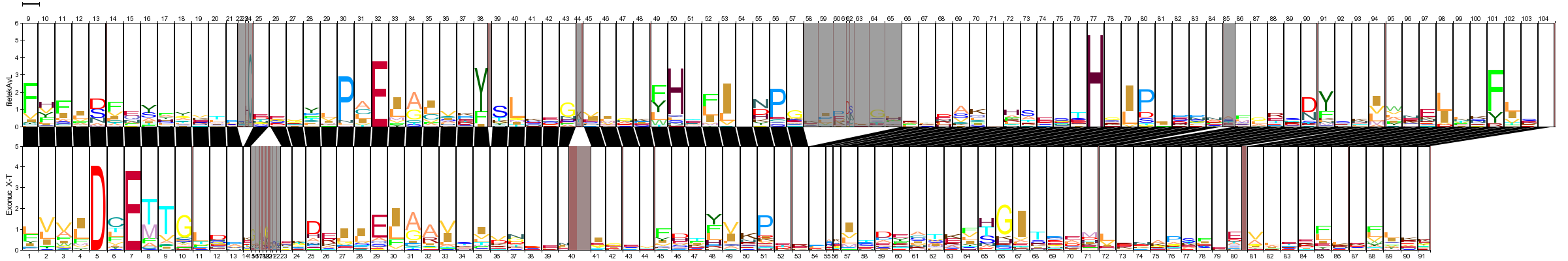


B: Profile-profile alignment between MAEL (above) and DEAD helicase (Pfam: PF00270) (bottom) domains by the logomat-p program.


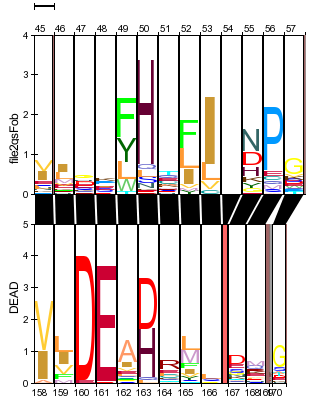

Supplement: Additional File 6 — Profile-profile alignment among MAEL, DnaQ (Exonuc X-T, Pfam: PF00929), and DEAD (Pfam: PF00270) domains by the logomat-p program. [file 1745-6150-3-48-S6.doc]
